# MHD Convective Flow of Jeffrey Fluid Due to a Curved Stretching Surface with Homogeneous-Heterogeneous Reactions

**DOI:** 10.1371/journal.pone.0161641

**Published:** 2016-09-01

**Authors:** Maria Imtiaz, Tasawar Hayat, Ahmed Alsaedi

**Affiliations:** 1 Department of Mathematics, Quaid-I-Azam University, 45320, Islamabad, 44000, Pakistan; 2 Nonlinear Analysis and Applied Mathematics (NAAM) Research Group, Department of Mathematics, Faculty of Science, King Abdulaziz University, 80203, Jeddah, 21589, Saudi Arabia; Tianjin University, CHINA

## Abstract

This paper looks at the flow of Jeffrey fluid due to a curved stretching sheet. Effect of homogeneous-heterogeneous reactions is considered. An electrically conducting fluid in the presence of applied magnetic field is considered. Convective boundary conditions model the heat transfer analysis. Transformation method reduces the governing nonlinear partial differential equations into the ordinary differential equations. Convergence of the obtained series solutions is explicitly discussed. Characteristics of sundry parameters on the velocity, temperature and concentration profiles are analyzed by plotting graphs. Computations for pressure, skin friction coefficient and surface heat transfer rate are presented and examined. It is noted that fluid velocity and temperature through curvature parameter are enhanced. Increasing values of Biot number correspond to the enhancement in temperature and Nusselt number.

## 1. Introduction

The study of non-Newtonian fluids has gained special focus of the recent researchers and engineers. Such motivation of the researchers is due to various applications of non-Newtonian fluids in technology and industrial areas. Unlike the viscous materials, the non-Newtonian fluids cannot be explained using well known Navier-Stokes theory. A single constitutive relationship cannot describe the characteristics of non-Newtonian liquids. The facts of non-Newtonian fluids are distinct than the viscous materials. The order of differential system in non-Newtonian fluid situation is higher than the viscous material. There are many proposed models of non-Newtonian fluids with diverse properties. These fluids in general have been classified into three categories known as the rate, the differential and the integral types. The most common and simplest model of non-Newtonian fluids is Jeffrey fluid. Such fluid has time derivative instead of convected derivative. Aspects of retardation and relaxation times are described by this fluid model. MHD flow of Jeffrey fluid in a cylindrical tube has been studied by Tripathi et al. [1]. Influences of slip and heat transfer on MHD peristaltic flow of Jeffrey fluid have been examined by Das [2]. Variable thermal conductivity of Jeffrey fluid in presence of thermal jump has been analyzed by Hamad et al. [3]. Shehzad et al. [4] presented the nonlinear thermal radiation effect in three dimensional flow of Jeffrey nanofluid. Ellahi and Hussain [5] examined slip feature in flow of Jeffrey fluid. Hayat et al. [6] studied stagnation point flow of Jeffrey nanofluid in presence of Newtonian heating. Flow of Jeffrey fluid due to oscillation of disks has been studied by Reddy et al. [7]. Farooq et al. [8] analyzed MHD flow of Jeffrey fluid in presence of Newtonian heating.

Many chemically reacting systems involve homogeneous-heterogeneous reactions for example in biochemical systems, combustion and catalysis. The correlation between homogeneous and heterogeneous reactions is very complex. Some of the reactions have the ability to proceed very slowly or not at all except in the presence of a catalyst. Fog formation and dispersion, food processing, ceramics and polymer production, hydrometallurgical industry etc. show obvious involvement of chemical reaction. Merkin [9] studied homogeneous-heterogeneous reactions in flow of viscous fluid over a flat plate. He considered homogeneous reaction for cubic autocatalysis and heterogeneous reaction on the catalyst surface. It is shown that surface reaction dominants near the plate. Homogenous-heterogeneous reactions with equal diffusivities have been examined by Chaudhary and Merkin [10]. Homogeneous-heterogeneous reactions in stretched flow of viscous fluid have been investigated by Bachok et al. [11]. Khan and Pop [12] presented stretched flow of viscoelastic fluid in presence of homogeneous-heterogeneous reactions. Shaw et al. [13] studied homogeneous-heterogeneous reactions in flow of micropolar fluid. Homogeneous-heterogeneous reactions in nanofluid flow over a permeable stretching surface have been analyzed by Kameswaran et al. [14]. Hayat et al. [15] investigated three dimensional flow of nanofluid in presence of second order slip velocity and homogeneous—heterogeneous reactions. Hayat et al. [16] also examined Cattaneo-Christov heat flux in MHD flow of Oldroyd-B fluid. Here homogeneous-heterogeneous reactions are considered.

Two-phase flow has wide applications in many industrial processes such as natural gas networks, spray processes, lubrication and nuclear reactor cooling. Main difference between single phase flow and multiphase flow is the existence of flow pattern which indicates a flow situation uniquely defined by the temporal and spatial distribution of the two immiscible phases. Three typical gas-liquid flow patterns are bubble flow, slug flow and churn flow. Research interests in the characterization of flow patterns lie on the fact that different flow patterns have distinct nonlinear dynamical properties. The recent advances in the study of multiphase flow are presented by Gao et al. [17–20].

Fluid flow by a stretching surface has promising applications in engineering and industrial processes such as in paper production, manufacture of foods, glass fiber, drawing of wires and plastic films, liquid films in condensation process, crystal growing, manufacturing and extraction of polymer and rubber sheets etc. Flow caused by stretching of a sheet has been examined by Crane [21]. After that stretched flow problems under different configurations have been examined by several researchers. Cortell [22] studied radiative nonlinear heat transfer in flow over a stretching sheet. Hsiao [23, 24] presented mixed convection effect in MHD flow of viscoelastic fluid past a stretching sheet with ohmic dissipation. Hsiao [25] examined MHD mixed convection for viscoelastic fluid past a porous wedge. Slip effect in stretched flow of nanofluid have been studied by Malvandi et al. [26]. Hsiao [27] investigated MHD stagnation point flow of nanofluid over a stretching sheet with mixed convection and partial slip. Sheikholeslami et al. [28] discussed effect of thermal radiation in magnetohydrodynamic nanofluid flow over a stretching sheet. Lin et al. [29] analyzed flow of pseudo-plastic nanoliquid over a stretching surface. In all these articles flat sheet is stretched and Cartesian coordinate system is used for mathematical modeling. Sajid et al. [30] provided fluid flow due to curved stretching sheet. They used curvilinear coordinate system in order to obtain the governing equations. They found that pressure is not negligible inside the boundary layer as in the case of a flat stretching sheet. Naveed et al. [31] studied MHD flow by a curved stretching surface. Time-dependent fluid flow due to curved stretching/shrinking surface has been presented by Rosca and Pop [32]. Radiative flow of nanofluid by a curved stretching surface with partial slip has been examined by Abbas et al. [33].

Main objective of present study is to extend the flow analysis of Sajid et al. [30] into following directions. Firstly, to model flow analysis for Jeffrey fluid. Secondly to predict the influence of homogeneous-heterogeneous reactions. Thirdly to examine heat transfer analysis in the presence of convective boundary conditions. Series solutions of present problem are computed by homotopy analysis method (HAM) [34–42]. The behaviors of different parameters on the physical quantities have been examined. Pressure, surface drag force and heat transfer rate are also studied. We hope that this study will lead to further investigations in future for various flow geometries and different flow models.

## 2. Model development

Consider two-dimensional flow of Jeffrey fluid induced by a curved stretching sheet at *r* = *R*. Stretching of sheet is taken in the *x* – direction with velocity *u* = *u*_*w*_. A magnetic field of strength *B*_0_ is applied in the *r* – direction. Also the bottom surface of sheet is heated by convection from a hot fluid at temperature *T*_*f*_ while ambient fluid temperature is *T*_∞_. Homogeneous-heterogeneous reactions of two chemical species *A* and *B* are considered. For cubic autocatalysis, the homogeneous reaction is

$$A+2B\to 3B,\;\text{rate}=k_{c}ab^{2},$$ (1)

while heterogeneous reaction on the catalyst surface is

$$A\to B,\;\text{rate}=k_{s}a,$$ (2)

where rate constants are defined by *k*_*c*_ and *k*_*s*_ and the chemical species *A* and *B* have concentrations *a* and *b*. Governing equations of present boundary layer flow problem are

$$(r+R)\frac{\partial v}{\partial r}+v+R\frac{\partial u}{\partial x}=0,$$ (3)

$$\frac{u^{2}}{r+R}=-\frac{1}{\rho }\frac{\partial p}{\partial r},$$ (4)

$$\begin{array}{l} v\frac{\partial u}{\partial r}+\frac{Ru}{r+R}\frac{\partial u}{\partial x}+\frac{uv}{r+R}=-\frac{1}{\rho }\frac{R}{r+R}\frac{\partial p}{\partial x}+\frac{\nu }{1+\lambda_{1}}[\frac{\partial^{2}u}{\partial r^{2}}+\frac{1}{r+R}\frac{\partial u}{\partial r}-\frac{u}{{\left(r+R\right)}^{2}}\\ \;+\lambda_{2}(\frac{\partial v}{\partial r}\frac{\partial^{2}u}{\partial r^{2}}+v\frac{\partial^{3}u}{\partial r^{3}}+\frac{R}{r+R}\frac{\partial u}{\partial r}\frac{\partial^{2}u}{\partial x\partial r}+\frac{R}{r+R}u\frac{\partial^{3}u}{\partial x\partial r^{2}}\\ \;+\frac{1}{r+R}v\frac{\partial^{2}u}{\partial r^{2}}-\frac{R}{{\left(r+R\right)}^{2}}\frac{\partial u}{\partial r}\frac{\partial u}{\partial x}-\frac{1}{r+R}\frac{\partial v}{\partial r}\frac{\partial u}{\partial r}\\ \;+\frac{1}{{\left(r+R\right)}^{2}}u\frac{\partial v}{\partial r})]-\frac{\sigma B_{0}^{2}u}{\rho }, \end{array}$$ (5)

$$v\frac{\partial T}{\partial r}+\frac{Ru}{r+R}\frac{\partial T}{\partial x}=\alpha^{*}\left(\frac{\partial^{2}T}{\partial r^{2}}+\frac{1}{r+R}\frac{\partial T}{\partial r}\right),$$ (6)

$$v\frac{\partial a}{\partial r}+\frac{Ru}{r+R}\frac{\partial a}{\partial x}=D_{A}\left(\frac{\partial^{2}a}{\partial r^{2}}+\frac{1}{r+R}\frac{\partial a}{\partial r}\right)-k_{c}ab^{2},$$ (7)

$$v\frac{\partial b}{\partial r}+\frac{Ru}{r+R}\frac{\partial b}{\partial x}=D_{B}\left(\frac{\partial^{2}b}{\partial r^{2}}+\frac{1}{r+R}\frac{\partial b}{\partial r}\right)+k_{c}ab^{2},$$ (8)

with boundary conditions

$$\begin{array}{l} \;u=u_{w}=cx,\;v=0,\;k_{f}\frac{\partial T}{\partial r}=h(T_{f}-T),\;D_{A}\frac{\partial a}{\partial r}=k_{s}a,\;D_{B}\frac{\partial b}{\partial r}=-k_{s}a\;\text{at}\;r=0,\\ u\to 0,\;\frac{\partial u}{\partial r}\to 0,\;T\to T_{\infty },\;a\to a_{0},\;b\to 0\;\text{as}\;r\to \infty , \end{array}$$ (9)

where the velocity components in (*r*,*x*) direction are (*v*,*u*) respectively, *p* denotes the pressure, *ρ* the density, *σ* the electrical conductivity, *ν* the kinematic viscosity, *c* > 0 the stretching constant, *λ*_1_ the ratio of relaxation to retardation times, *λ*_2_ the retardation time, *h* the convective heat transfer coefficient, *T* the temperature, *k*_*f*_ the thermal conductivity and α* the thermal diffusivity.

Using the following transformations

$$\begin{array}{l} \;u=cxf^{\prime }(\xi ),\;v=-\frac{R}{r+R}\sqrt{c\nu }f(\xi ),\;\xi =\sqrt{\frac{c}{\nu }}r,\;p=\rho c^{2}x^{2}P(\xi ),\\ \theta (\xi )=\frac{T-T_{\infty }}{T_{f}-T_{\infty }},\;a=a_{0}\Phi (\xi ),\;b=a_{0}g(\xi ). \end{array}$$ (10)

Eq (3) is satisfied automatically and Eqs 4–8 can be reduced as follows:

$$\frac{\partial P}{\partial \xi }=\frac{f^{\prime 2}}{\xi +K}$$ (11)

$$\begin{array}{l} \frac{2K}{\xi +K}P=\frac{K}{\xi +K}ff^{\prime \prime }+\frac{K}{{\left(\xi +K\right)}^{2}}ff^{\prime }-\frac{K}{\xi +K}f^{\prime 2}+\frac{1}{1+\lambda_{1}}[f^{\prime \prime \prime }+\frac{1}{\xi +K}f^{\prime \prime }\\ \;-\frac{1}{{\left(\xi +K\right)}^{2}}f^{\prime }+\lambda \{\frac{K}{\xi +K}f^{\prime \prime 2}-\frac{K}{\xi +K}ff^{iv}-\frac{K}{{\left(\xi +K\right)}^{3}}ff^{\prime \prime }\\ \;+\frac{K}{{\left(\xi +K\right)}^{4}}ff^{\prime }-\frac{K}{{\left(\xi +K\right)}^{3}}f^{\prime 2}\}]-Mf^{\prime }, \end{array}$$ (12)

$$\frac{1}{\mathrm{Pr}}\left(\theta^{\prime \prime }+\frac{1}{\xi +K}\theta^{\prime }\right)+\frac{K}{\xi +K}f\theta^{\prime }=0,$$ (13)

$$\frac{1}{Sc}\left(\Phi^{\prime \prime }+\frac{1}{\xi +K}\Phi^{\prime }\right)+\frac{K}{\xi +K}f\Phi^{\prime }-k_{1}\Phi g^{2}=0,$$ (14)

$$\frac{\delta }{Sc}\left(g^{\prime \prime }+\frac{1}{\xi +K}g^{\prime }\right)+\frac{K}{\xi +K}fg^{\prime }+k_{1}\Phi g^{2}=0,$$ (15)

$$\begin{array}{l} \;f^{\prime }(0)=1,\;f(0)=0,\;\theta^{\prime }(0)=-\gamma_{1}\left[1-\theta (0)\right],\;\Phi^{\prime }(0)=k_{2}\Phi (0),\;\delta g^{\prime }(0)=-k_{2}\Phi (0),\\ f^{\prime }(\infty )\to 0,\;f^{\prime \prime }(\infty )\to 0,\;\theta (\infty )\to 0,\;\Phi (\infty )\to 1,\;g(\infty )\to 0, \end{array}$$ (16)

where $M=\sigma B_{0}^{2}/\rho c$ is the Hartman number, *λ* = *λ*_2_*c* is the Deborah number, $K=R\sqrt{c/\nu }$ is the curvature parameter, Pr = *ν* / *α* is the Prandtl number, *δ* = *D*_*B*_ / *D*_*A*_ is the ratio of diffusion coefficient, *Sc* = *ν* / *D*_*A*_ is the Schmidt number, $k_{1}=a_{0}^{2}k_{c}/c$ is the homogeneous reaction strength, $k_{2}=k_{s}\sqrt{\nu }/D_{A}\sqrt{c}$ is the heterogeneous reaction strength and $\gamma_{1}=h\sqrt{\nu }/k\sqrt{c}$ is the Biot number.

Now eliminating pressure *P* between Eqs 11 and 12, we obtain

$$\begin{array}{l} f^{iv}+\frac{2}{\xi +K}f^{\prime \prime \prime }+\frac{1}{{\left(\xi +K\right)}^{3}}f^{\prime }-\frac{1}{{\left(\xi +K\right)}^{2}}f^{\prime \prime }+\lambda (\frac{2K}{\xi +K}f^{\prime \prime }f^{\prime \prime \prime }-\frac{K}{\xi +K}f^{\prime }f^{iv}-\frac{K}{\xi +K}ff^{v}\\ +\frac{3K}{{\left(\xi +K\right)}^{4}}ff^{\prime \prime }-\frac{K}{{\left(\xi +K\right)}^{3}}ff^{\prime \prime \prime }-\frac{3K}{{\left(\xi +K\right)}^{3}}f^{\prime }f^{\prime \prime }-\frac{3K}{{\left(\xi +K\right)}^{5}}ff^{\prime }+\frac{3K}{{\left(\xi +K\right)}^{4}}f^{\prime 2})\\ +(1+\lambda_{1})\left(\frac{K}{\xi +K}(ff^{\prime \prime \prime }-f^{\prime }f^{\prime \prime })+\frac{K}{{\left(\xi +K\right)}^{2}}(ff^{\prime \prime }-f^{\prime 2})-\frac{K}{{\left(\xi +K\right)}^{3}}ff^{\prime }-Mf^{\prime \prime }-\frac{M}{\xi +K}f^{\prime }\right)=0 \end{array}$$ (17)

with boundary conditions

$$f^{\prime }(0)=1,\;f(0)=0,\;f^{\prime }(\infty )\to 0,\;f^{\prime \prime }(\infty )\to 0.$$ (18)

Pressure can now be determined from Eq 12 as

$$\begin{array}{l} P=\frac{1}{2}ff^{\prime \prime }+\frac{1}{2(\xi +K)}ff^{\prime }-\frac{1}{2}f^{\prime 2}+\frac{1}{1+\lambda_{1}}[\frac{\xi +K}{2K}f^{\prime \prime \prime }\;\text{-}\\ \;+\frac{1}{2K}f^{\prime \prime }-\frac{1}{2K(\xi +K)}f^{\prime }+\lambda (\frac{1}{2}f^{\prime \prime 2}-\frac{1}{2}ff^{iv}-\frac{1}{2{\left(\xi +K\right)}^{2}}ff^{\prime \prime }\\ \;+\frac{1}{2{\left(\xi +K\right)}^{3}}ff^{\prime }-\frac{1}{2{\left(\xi +K\right)}^{2}}f^{\prime 2})]-M\frac{\xi +K}{2K}f^{\prime }. \end{array}$$ (19)

Here it is assumed that both chemical species have equal diffusion coefficients *D*_*A*_ and *D*_*B*_, i.e. *δ* = 1 and thus

Φ(ξ)+g(ξ)=1. (20)

Now Eqs 14 and 15 yield

$$\frac{1}{Sc}\left(\Phi^{\prime \prime }+\frac{1}{\xi +K}\Phi^{\prime }\right)+\frac{K}{\xi +K}f\Phi^{\prime }-k_{1}\Phi {\left(1-\Phi \right)}^{2}=0,$$ (21)

with the boundary conditions

$$\Phi^{\prime }(0)=k_{2}\Phi (0),\;\Phi (\infty )\to 1.$$ (22)

Skin friction coefficient *C*_*f*_ and Nusselt number *Nu* are

$$C_{f}=\frac{\tau_{rx}}{\frac{1}{2}\rho u_{w}^{2}},\;Nu=\frac{xq_{w}}{k_{f}\left(T_{w}-T_{\infty }\right)},$$ (23)

where *τ*_*rx*_ represents surface shear stress and *q*_*w*_ the wall heat flux which are given by

$$\begin{array}{l} \tau_{rx}=\frac{\mu }{1+\lambda_{1}}\left[\frac{\partial u}{\partial r}-\frac{u}{r+R}+\lambda_{2}\{\frac{Ru}{r+R}\frac{\partial^{2}u}{\partial x\partial r}-\frac{Ru}{{\left(r+R\right)}^{2}}\frac{\partial u}{\partial x}\right]\\ \;{+v\frac{\partial^{2}u}{\partial r^{2}}-\frac{v}{r+R}\frac{\partial u}{\partial r}+\frac{uv}{{\left(r+R\right)}^{2}}\}]}_{r=0}, \end{array}$$ (24)

$$q_{w}=-{k_{f}\frac{\partial T}{\partial r}|}_{r=0}.$$ (25)

Finally, we have

$$\frac{1}{2}C_{f}{\left(Re_{x}\right)}^{1/2}=\frac{1}{1+\lambda_{1}}[\left[f^{\prime \prime }(0)-\frac{f^{\prime }(0)}{K}+\lambda \left(f^{\prime }(0)f^{\prime \prime }(0)-\frac{1}{K^{2}}{\left[f^{\prime }(0)\right]}^{2}\right)\right],$$ (26)

$$Nu{\left(Re_{x}\right)}^{-1/2}=-\theta^{\prime }(0),$$ (27)

where local Reynolds number is defined as Re_*x*_ = *cx*^2^ / *ν*.

## 3. Homotopic solutions

### 3.1. Zero^*th*^-order deformation problems

Auxiliary functions **H**_*f*_, **H**_*θ*_ and **H**_Φ_, linear operators *L*_1_, *L*_2_ and *L*_3_ and the initial guesses *f*_0_(*ξ*), *θ*_0_(*ξ*) and Φ_0_(*ξ*) are taken in the forms

$$H_{f}=e^{-2\xi },\;H_{\theta }=e^{-2\xi },\;H_{\Phi }=e^{-2\xi },$$ (28)

$$L_{1}=f^{iv}-5f^{\prime \prime }+4f,\;L_{2}=\theta^{\prime \prime }-\theta ,\;L_{3}=\Phi^{\prime \prime }-\Phi ,$$ (29)

$$f_{0}(\xi )=e^{-\xi }-e^{-2\xi },\;\theta_{0}(\xi )=\frac{\gamma_{1}}{1+\gamma_{1}}e^{-\xi },\;\Phi_{0}(\xi )=1-\frac{1}{2}e^{-k_{2}\xi },$$ (30)

subject to the properties

$$L_{1}[c_{1}e^{\xi }+c_{2}e^{-\xi }+c_{3}e^{2\xi }+c_{4}e^{-2\xi }]=0,$$ (31)

$$L_{2}[c_{5}e^{\xi }+c_{6}e^{-\xi }]=0,$$ (32)

$$L_{3}[c_{7}e^{\xi }+c_{8}e^{-\xi }]=0,$$ (33)

in which *c*_*i*_ (*i* = 1–8) are the constants.

If ℏ_*f*_, ℏ_*θ*_ and ℏ_Φ_ are nonzero auxiliary parameters and *p* ∈ [0,1] denotes embedding parameter then the zeroth order deformation problems are as follows:

$$(1-p)L_{1}[F(\xi ;\;p)-f_{0}(\xi )]=p\hbar_{f}H_{f}N_{f}[F(\xi ;\;p)],$$ (34)

$$(1-p)L_{2}[\Theta (\xi ;\;p)-\theta_{0}(\xi )]=p\hbar_{\theta }H_{\theta }N_{\theta }[\Theta (\xi ;\;p),\;F(\xi ;\;p)],$$ (35)

$$(1-p)L_{3}[\phi (\xi ;\;p)-\Phi_{0}(\xi )]=p\hbar_{\Phi }H_{\Phi }N_{\Phi }[\phi (\xi ;\;p),\;F(\xi ;\;p)],$$ (36)

$$\begin{array}{l} \;F^{\prime }(0;\;p)=1,\;F(0;\;p)=0,\;\Theta^{\prime }(0;\;p)=-\gamma_{1}[1-\Theta (0;\;p)],\;\phi^{\prime }(0;\;p)=k_{2}\phi (0;\;p),\\ F^{\prime }(\infty ;\;p)=0,\;F^{\prime \prime }(\infty ;\;p)=0,\;\Theta (\infty ;\;p)=0,\;\phi (\infty ,\;p)=1. \end{array}$$ (37)

Nonlinear operators are

$$\begin{array}{l} N_{f}=\frac{\partial^{4}F(\xi ;\;p)}{\partial \xi^{4}}+\frac{2}{\xi +K}\frac{\partial^{3}F(\xi ;\;p)}{\partial \xi^{3}}+\frac{1}{{\left(\xi +K\right)}^{3}}\frac{\partial F(\xi ;\;p)}{\partial \xi }-\frac{1}{{\left(\xi +K\right)}^{2}}\frac{\partial^{2}F(\xi ;\;p)}{\partial \xi^{2}}\\ \;+\lambda \frac{K}{\xi +K}(2\frac{\partial^{2}F(\xi ;\;p)}{\partial \xi^{2}}\frac{\partial^{3}F(\xi ;\;p)}{\partial \xi^{3}}-\frac{\partial F(\xi ;\;p)}{\partial \xi }\frac{\partial^{4}F(\xi ;\;p)}{\partial \xi^{4}}-F(\xi ;\;p)\frac{\partial^{5}F(\xi ;\;p)}{\partial \xi^{5}}\\ \;+\frac{3}{{\left(\xi +K\right)}^{3}}F(\xi ;\;p)\frac{\partial^{2}F(\xi ;\;p)}{\partial \xi^{2}}-\frac{1}{{\left(\xi +K\right)}^{2}}F(\xi ;\;p)\frac{\partial^{3}F(\xi ;\;p)}{\partial \xi^{3}}+\frac{3}{{\left(\xi +K\right)}^{3}}{\left(\frac{\partial F(\xi ;\;p)}{\partial \xi }\right)}^{2}\\ \;-\frac{3}{{\left(\xi +K\right)}^{4}}F(\xi ;\;p)\frac{\partial F(\xi ;\;p)}{\partial \xi }-\frac{3}{{\left(\xi +K\right)}^{2}}\frac{\partial F(\xi ;\;p)}{\partial \xi }\frac{\partial^{2}F(\xi ;\;p)}{\partial \xi^{2}})+(1+\lambda_{1})\frac{K}{\xi +K}\\ \;+(F(\xi ;\;p)\frac{\partial^{3}F(\xi ;\;p)}{\partial \xi^{3}}-\frac{\partial F(\xi ;\;p)}{\partial \xi }\frac{\partial^{2}F(\xi ;\;p)}{\partial \xi^{2}})+\frac{1}{\xi +K}F(\xi ;\;p)\frac{\partial^{2}F(\xi ;\;p)}{\partial \xi^{2}}\\ \;-\frac{1}{\xi +K}{\left(\frac{\partial F(\xi ;\;p)}{\partial \xi }\right)}^{2}-\frac{1}{{\left(\xi +K\right)}^{2}}F(\xi ;\;p)\frac{\partial F(\xi ;\;p)}{\partial \xi }-M\frac{\partial^{2}F(\xi ;\;p)}{\partial \xi^{2}}-\frac{M}{\xi +K}\frac{\partial F(\xi ;\;p)}{\partial \xi }), \end{array}$$ (38)

$$N_{\theta }=\frac{1}{\mathrm{Pr}}\left(\frac{\partial^{2}\Theta (\xi ;\;p)}{\partial \xi^{2}}+\frac{1}{\xi +K}\frac{\partial \Theta (\xi ;\;p)}{\partial \xi }\right)+\frac{K}{\xi +K}F(\xi ;\;p)\frac{\partial \Theta (\xi ;\;p)}{\partial \eta },$$ (39)

$$\begin{array}{l} N_{\Phi }=\frac{1}{Sc}\left(\frac{\partial^{2}\phi (\xi ;\;p)}{\partial \xi^{2}}+\frac{1}{\xi +K}\frac{\partial \phi (\xi ;\;p)}{\partial \xi }\right)+\frac{K}{\xi +K}F(\xi ;\;p)\frac{\partial \phi (\xi ;\;p)}{\partial \xi }\\ \;-k_{1}\phi (\xi ;\;p){\left(1-\phi (\xi ;\;p)\right)}^{2}. \end{array}$$ (40)

### 3.2. m^*th*^-order deformation problems

The mth order deformation problems are

$$L_{1}\left[f_{m}-\chi_{m}f_{m-1}\right]=\hbar_{f}R_{m}^{f},$$ (41)

$$L_{2}\left[\theta_{m}-\chi_{m}\theta_{m-1}\right]=\hbar_{\theta }R_{m}^{\theta },$$ (42)

$$L_{3}\left[\Phi_{m}-\chi_{m}\Phi_{m-1}\right]=\hbar_{\Phi }R_{m}^{\Phi },$$ (43)

$$\begin{array}{l} {\frac{\partial f_{m}}{\partial \xi }|}_{\xi =0}={f_{m}|}_{\xi =0}={\frac{\partial f_{m}}{\partial \xi }|}_{\xi \to \infty }=0,\\ {\frac{\partial \theta_{m}}{\partial \xi }|}_{\xi =0}-\gamma_{1}{\theta_{m}|}_{\xi =0}={\theta_{m}|}_{\xi \to \infty }=0,\\ {\frac{\partial \Phi_{m}}{\partial \xi }|}_{\xi =0}-k_{2}{\Phi_{m}|}_{\xi =0}={\Phi_{m}|}_{\xi \to \infty }=0, \end{array}$$ (44)

$$\begin{array}{l} R_{m}^{f}=f_{m-1}^{iv}+\frac{2}{\xi +K}f_{m-1}^{\prime \prime \prime }+\frac{1}{{\left(\xi +K\right)}^{3}}f_{m-1}^{\prime }-\frac{1}{{\left(\xi +K\right)}^{2}}f_{m-1}^{\prime \prime }+\lambda \sum_{k=0}^{m-1}(\frac{2K}{\xi +K}f_{m-1-k}^{\prime \prime }f_{k}^{\prime \prime \prime }\\ \;-\frac{K}{\xi +K}f_{m-1-k}^{\prime }f_{k}^{iv}-\frac{K}{\xi +K}f_{m-1-k}f_{k}^{v}+\frac{3K}{{\left(\xi +K\right)}^{4}}f_{m-1-k}f_{k}^{\prime \prime }-\frac{K}{{\left(\xi +K\right)}^{3}}f_{m-1-k}f_{k}^{\prime \prime \prime }\\ \;-\frac{3K}{{\left(\xi +K\right)}^{3}}f_{m-1-k}^{\prime }f_{k}^{\prime \prime }-\frac{3K}{{\left(\xi +K\right)}^{5}}f_{m-1-k}f_{k}^{\prime }+\frac{3K}{{\left(\xi +K\right)}^{4}}f_{m-1-k}^{\prime }f_{k}^{\prime })+(1+\lambda_{1})[\frac{K}{\xi +K}\\ \;\sum_{k=0}^{m-1}\left(f_{m-1-k}f_{k}^{\prime \prime \prime }-f_{m-1-k}^{\prime }f_{k}^{\prime \prime }+\frac{1}{\xi +K}(f_{m-1-k}f_{k}^{\prime \prime }-f_{m-1-k}^{\prime }f_{k}^{\prime })-\frac{1}{{\left(\xi +K\right)}^{2}}f_{m-1-k}f_{k}^{\prime }\right)\\ \;-Mf_{m-1}^{\prime \prime }-\frac{M}{\xi +K}f_{m-1}^{\prime }], \end{array}$$ (45)

$$R_{m}^{\theta }=\frac{1}{\mathrm{Pr}}\left(\theta_{m-1}^{\prime \prime }+\frac{1}{\xi +K}\theta_{m-1}^{\prime }\right)+\frac{K}{\xi +K}\sum_{k=0}^{m-1}f_{m-1-k}\theta_{k}^{\prime },$$ (46)

$$\begin{array}{l} R_{m}^{\Phi }=\frac{1}{Sc}\left(\Phi_{m-1}^{\prime \prime }+\frac{1}{\xi +K}\Phi_{m-1}^{\prime }\right)+\frac{K}{\xi +K}\sum_{k=0}^{m-1}f_{m-1-k}\Phi_{k}^{\prime }\\ \;-k_{1}\sum_{k=0}^{m-1}\left(\Phi_{m-1-l}\sum_{j=0}^{l}\Phi_{l-j}\Phi_{j}-2\Phi_{m-1-l}\Phi_{l}\right)-k_{1}\Phi_{m-1}, \end{array}$$ (47)

$$\chi_{m}=\{\begin{array}{l} 0,\;m\leq 1\\ 1,\;m>1 \end{array}.$$ (48)

The general solutions (*f*_*m*_, *θ*_*m*_, Φ_*m*_) comprising the special solutions $(f_{m}^{*},$ $\theta_{m}^{*},$ $\Phi_{m}^{*})$ are

$$f_{m}(\xi )=f_{m}^{*}(\xi )+c_{1}e^{\xi }+c_{2}e^{-\xi }+c_{3}e^{2\xi }+c_{4}e^{-2\xi },$$ (49)

$$\theta_{m}(\xi )=\theta_{m}^{*}(\xi )+c_{5}e^{\xi }+c_{6}e^{-\xi },$$ (50)

$$\Phi_{m}(\xi )=\Phi_{m}^{*}(\xi )+c_{7}e^{\xi }+c_{8}e^{-\xi },$$ (51)

where the constants *c*_*i*_ (*i* = 1–8) through the boundary conditions (44) have the values

$$\begin{array}{l} c_{1}=c_{3}=c_{5}=c_{7}=0,\;c_{2}=-c_{4}-f_{m}^{*}(0),\;c_{4}={\frac{\partial \theta_{m}^{*}(\xi )}{\partial \xi }|}_{\xi =0}+f_{m}^{*}(0),\\ c_{6}=\frac{1}{1+\gamma_{1}}\left[{\frac{\partial \theta_{m}^{*}(\xi )}{\partial \xi }|}_{\xi =0}-\gamma_{1}\theta_{m}^{*}(0)\right],\;c_{8}=\frac{1}{1+k_{2}}\left[{\frac{\partial \Phi_{m}^{*}(\xi )}{\partial \xi }|}_{\xi =0}-k_{2}\Phi_{m}^{*}(0)\right]. \end{array}$$ (52)

## 4. Convergence analysis

**Fig 1:**
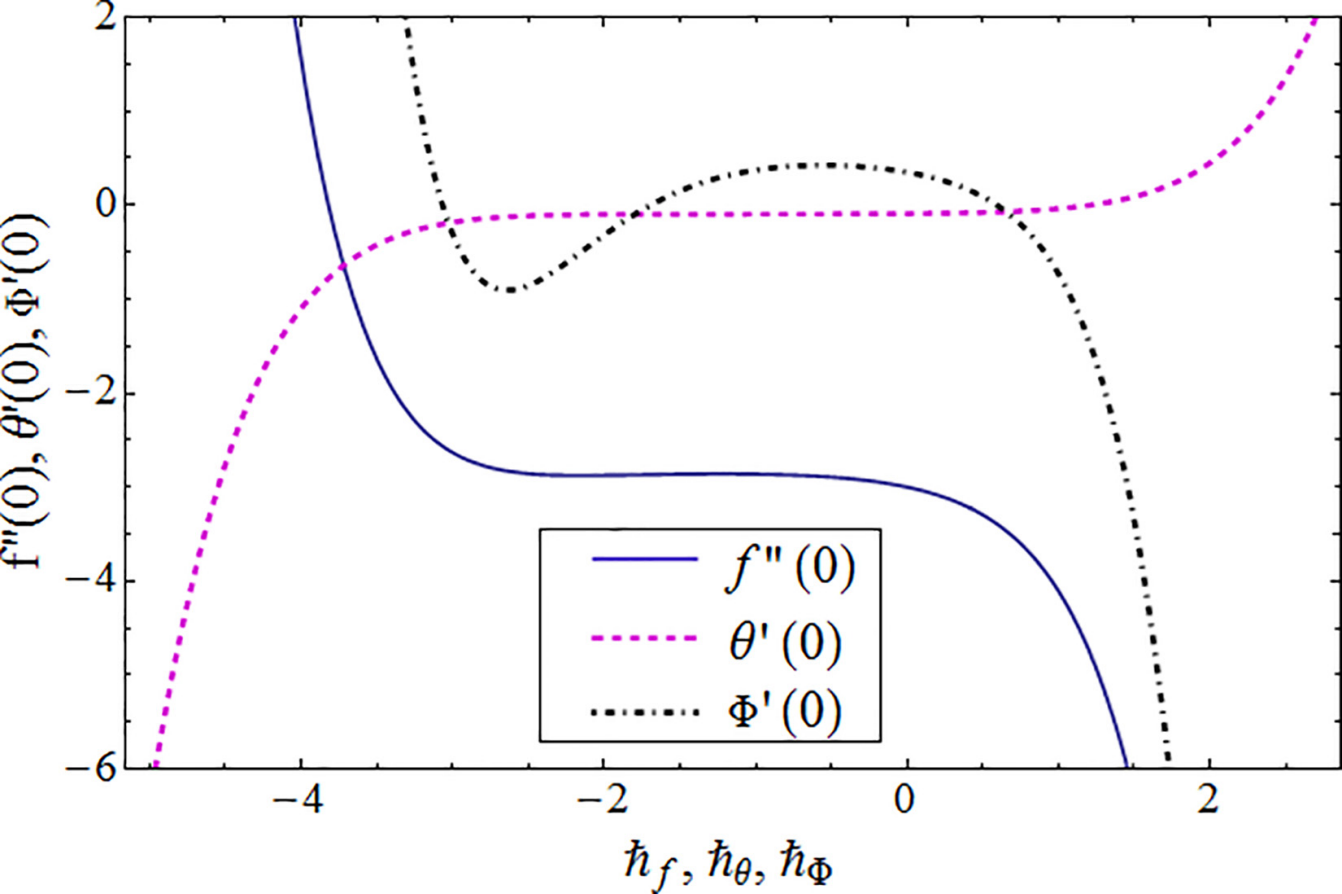
The ℏ–curves for *f*″(0), *θ*′(0) and Φ′(0) when *K* = 0.01, *λ*_1_ = 0.5, *λ* = 0.9, *γ*_1_ = 0.1, *M* = 0.3, Pr = 1, *Sc* = *k*_1_ = 0.9 and *k*_2_ = 0.7.

Homotopy analysis method (HAM) involves an embedding auxiliary parameter ℏ which gives the freedom to choose and adjust convergence region of series solutions. The ℏ–curves are plotted to obtain valid ranges of these parameters (see Fig 1). Allowed values of ℏ_*f*_, ℏ_*θ*_ and ℏ_Φ_ are −1.7 ≤ ℏ_*f*_ ≤ −0.9, −2 ≤ ℏ_*θ*_ ≤ −0.2 and −0.7 ≤ ℏ_Φ_ ≤ −0.3. Also HAM solutions converge when ℏ_*f*_ = −0.9, ℏ_*θ*_ = −1 and ℏ_Φ_ = −0.3 (Table 1).

**Table 1:** HAM solutions convergence when *K* = 0.01, *λ*_1_ = 0.5, *λ* = 0.9, *γ*_1_ = 0.1, *M* = 0.3, Pr = 1, *Sc* = *k*_1_ = 0.9 and *k*_2_ = 0.7.

Order of approximations	–*f*″(0)	–*θ*′(0)	Φ′(0)
1	12.956	0.09395	0.3557
5	2.879	0.09867	0.3773
8	2.865	0.09924	0.3925
10	2.865	0.09931	0.4022
15	2.865	0.09939	0.4167
20	2.865	0.09939	0.4179
26	2.865	0.09939	0.4184
30	2.865	0.09939	0.4184

## 5. Results and Discussion

In this section the effects of different parameters on the velocity, temperature and concentration fields are investigated through plots.

### 5.1. Dimensionless velocity profile

**Fig 2:**
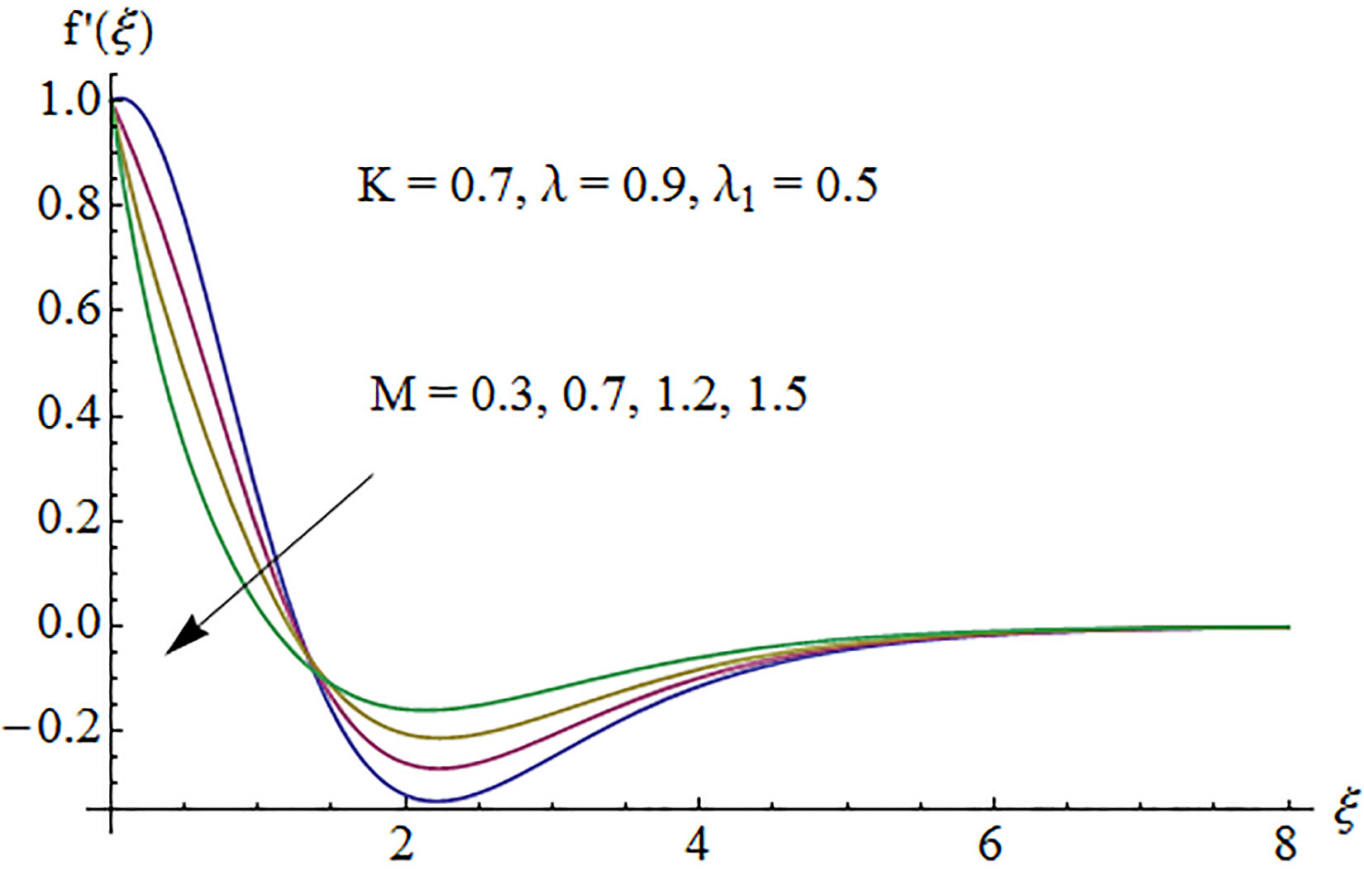
Impact of *M* on velocity.

**Fig 3:**
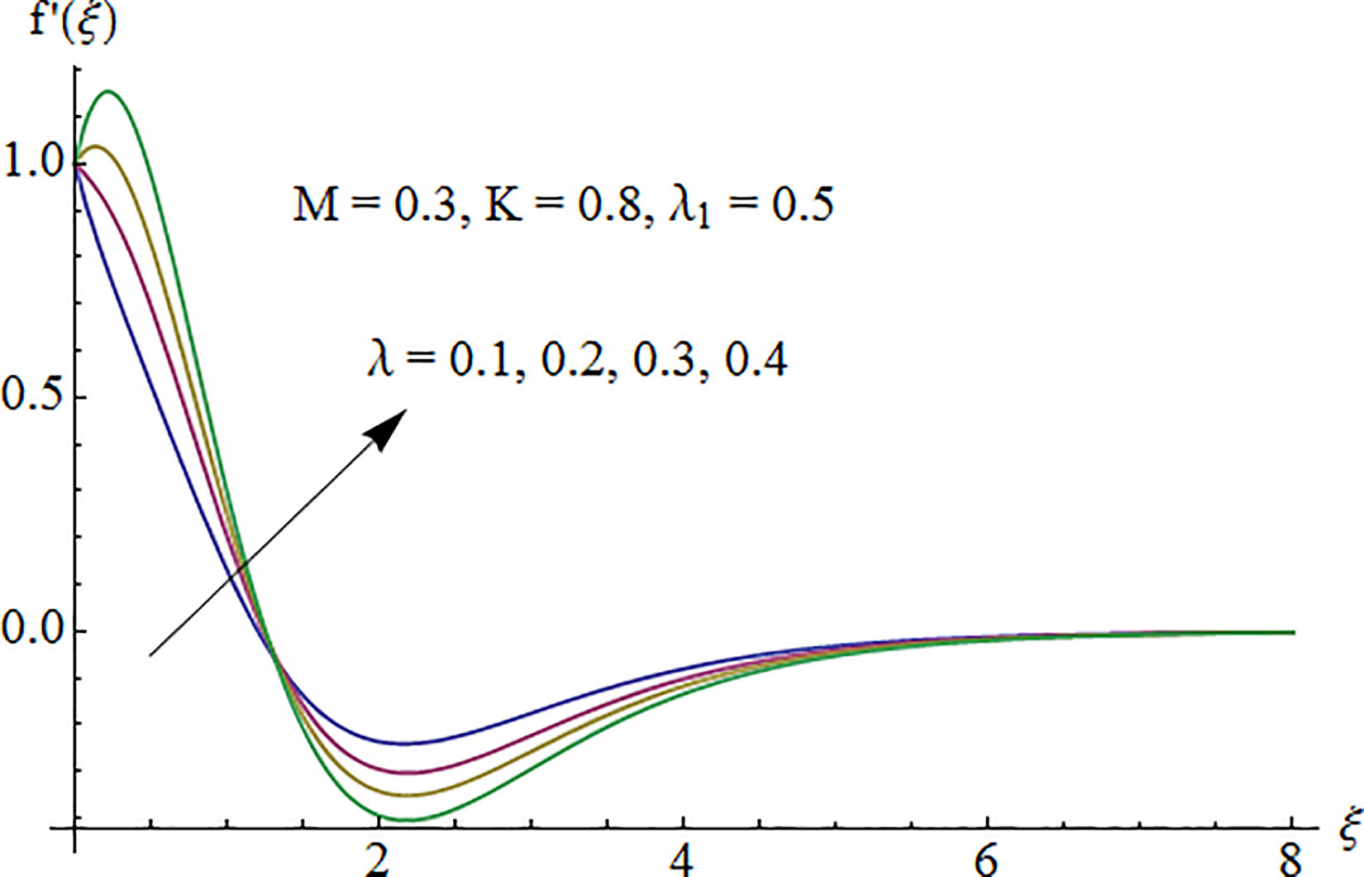
Impact of *λ* on velocity.

**Fig 4:**
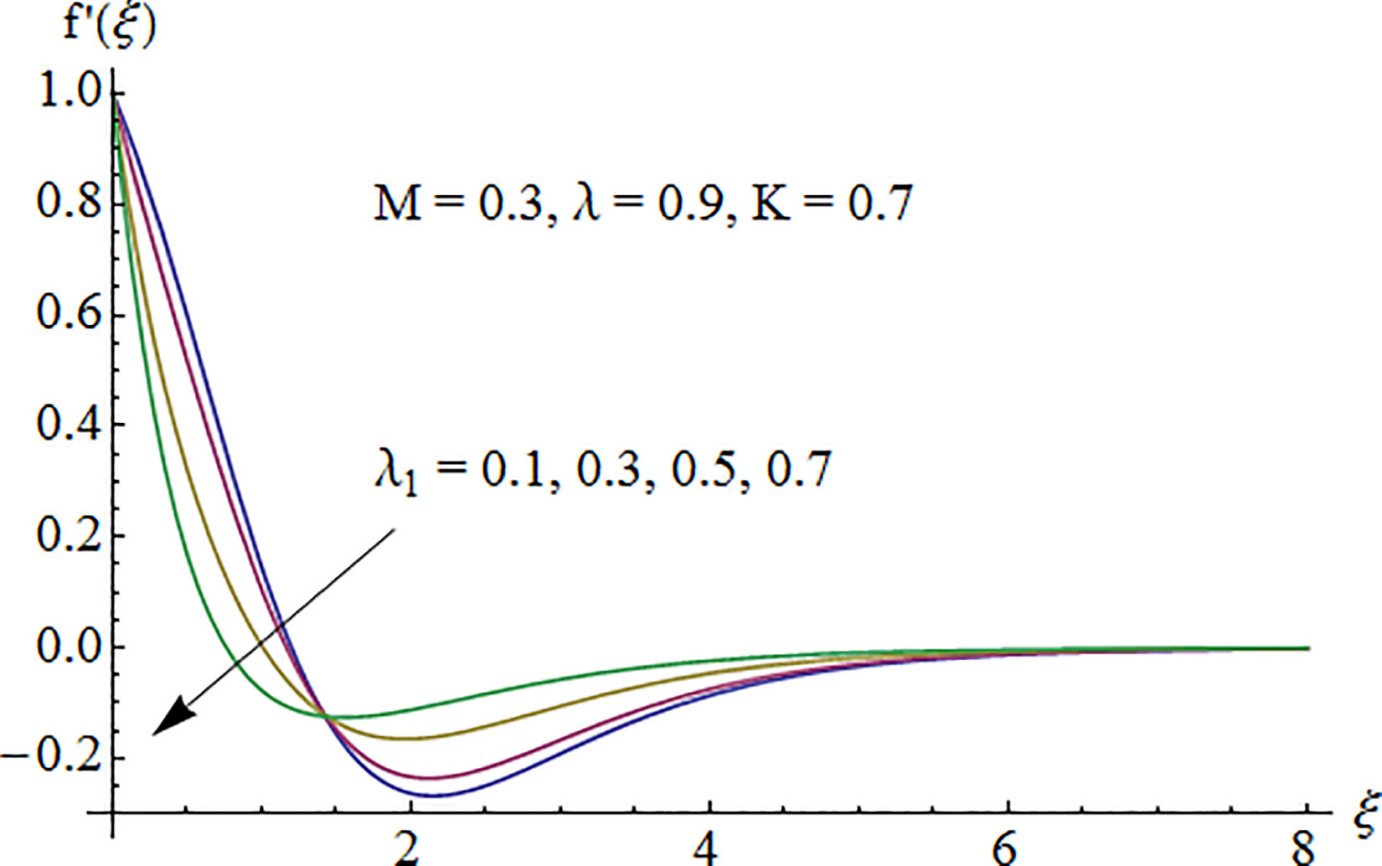
Impact of *λ*_1_ on velocity.

**Fig 5:**
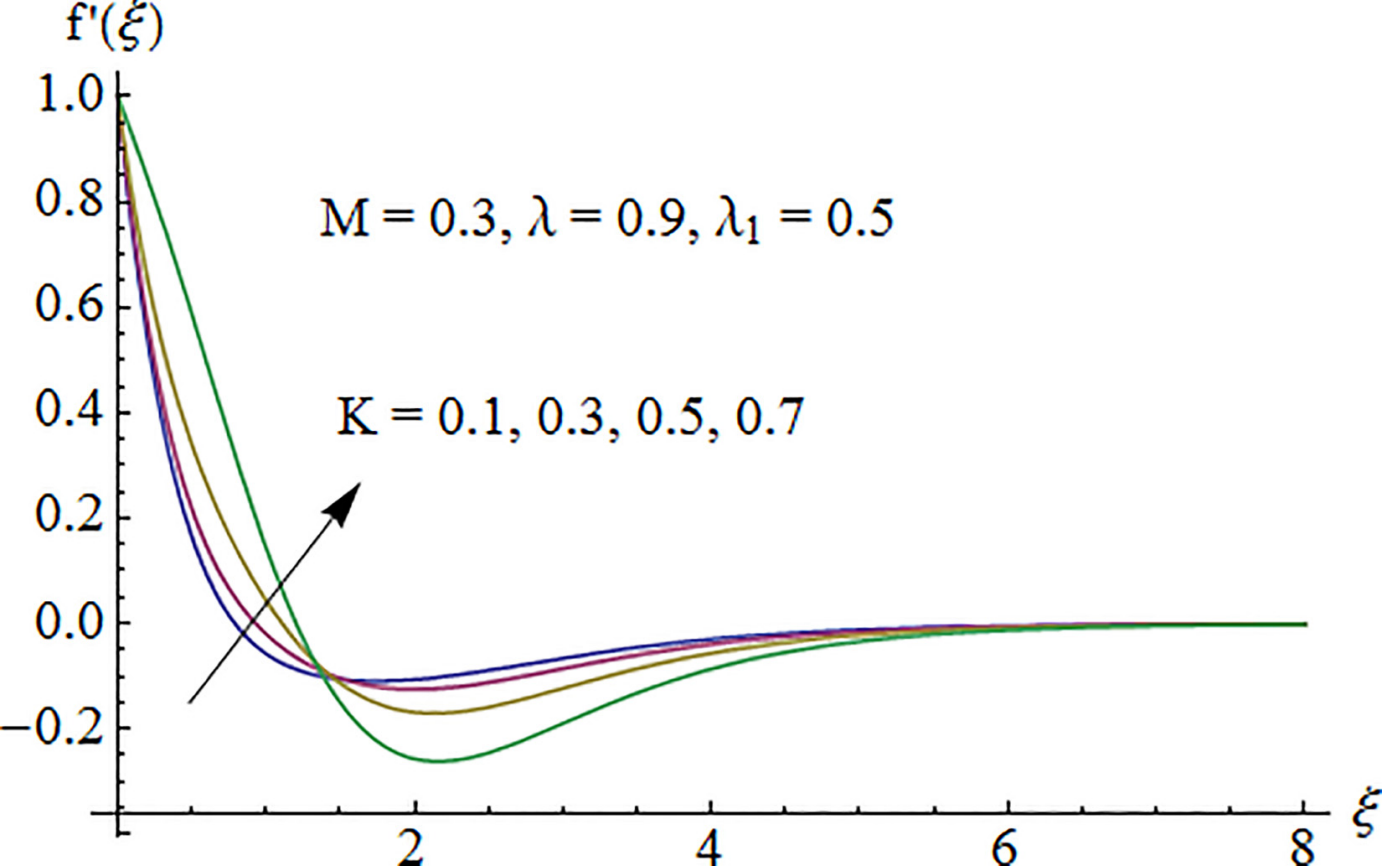
Impact of *K* on velocity.

Fig 2 depicts the variation of Hartman number *M* on the velocity distribution *f*′(*ξ*). An enhancement in the strength of magnetic field produces a resistive force which signifies the reduction of the fluid velocity. Here negative values of *f*′(*ξ*) indicate downward flow in the vertical direction. Fig 3 illustrates the behavior of Deborah number *λ* on the horizontal component of velocity *f*′(*ξ*). An increase in retardation time enhances elasticity. Since elasticity and viscosity effects are inversely proportional to each other so decrease in viscosity enhances the fluid velocity. Impact of *λ*_1_ on velocity profile *f*′(*ξ*) is depicted in Fig 4. An increase in *λ*_1_ corresponds to increase in relaxation time. It means particle needs much more time to come back from perturbed system to equilibrium system and consequently the fluid velocity decreases. Fig 5 shows impact of curvature parameter *K* on the velocity profile *f*′(*ξ*). Here increment in the magnitude of velocity profile is subjected to the enhanced values of *K*.

### 5.2. Dimensionless temperature profile

**Fig 6:**
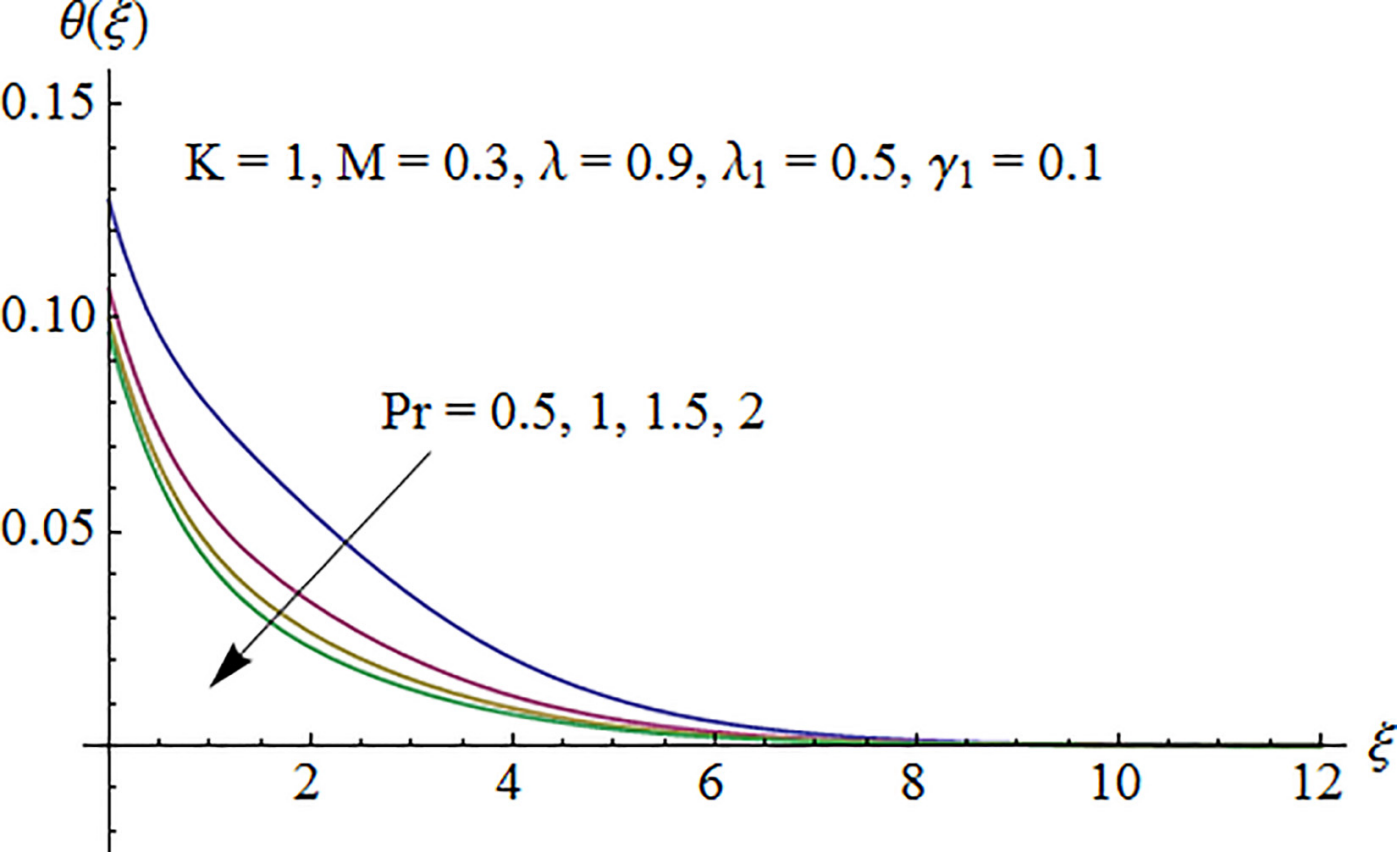
Impact of Pr on temperature.

**Fig 7:**
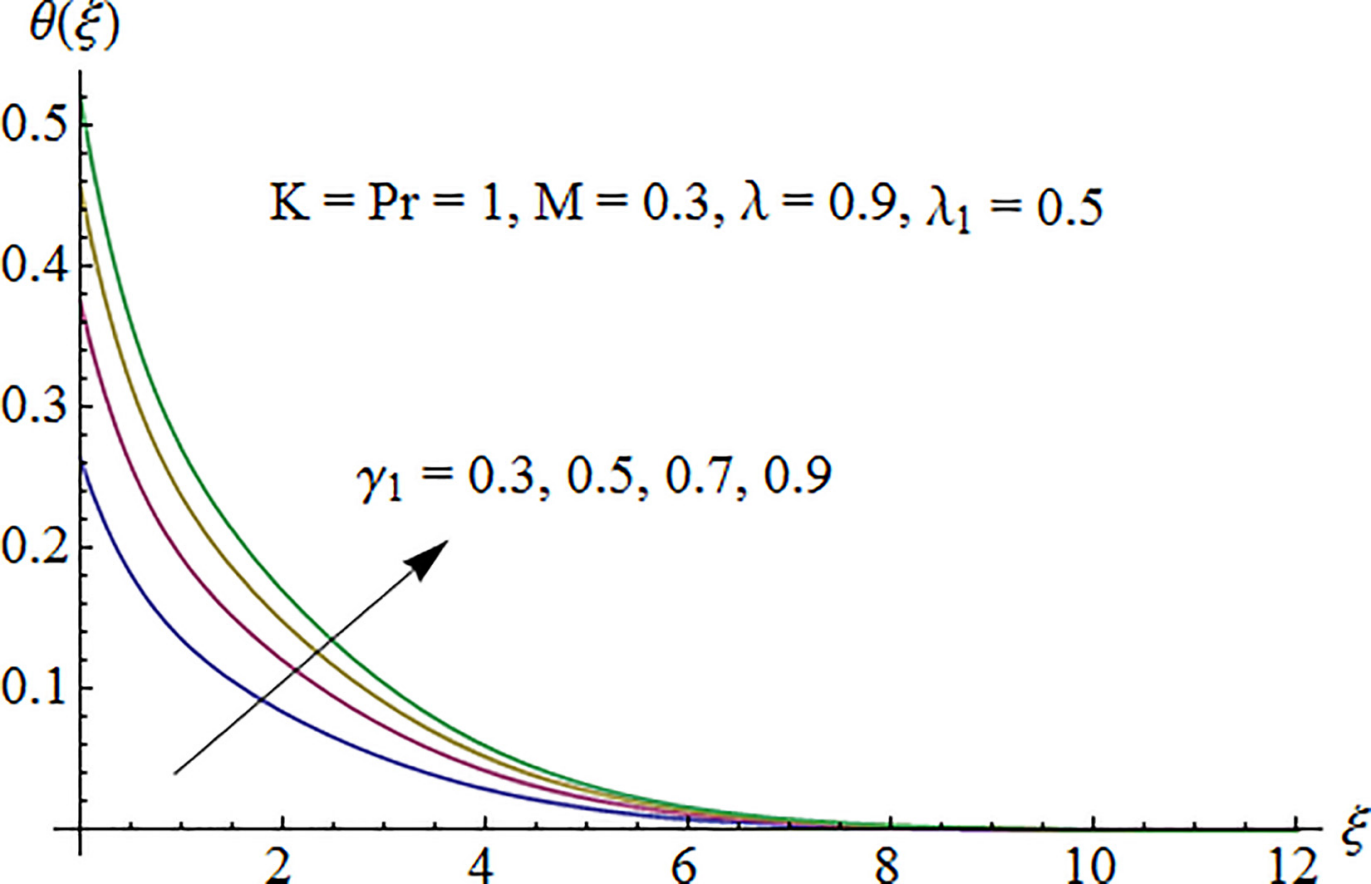
Impact of *γ*_1_ on temperature.

**Fig 8:**
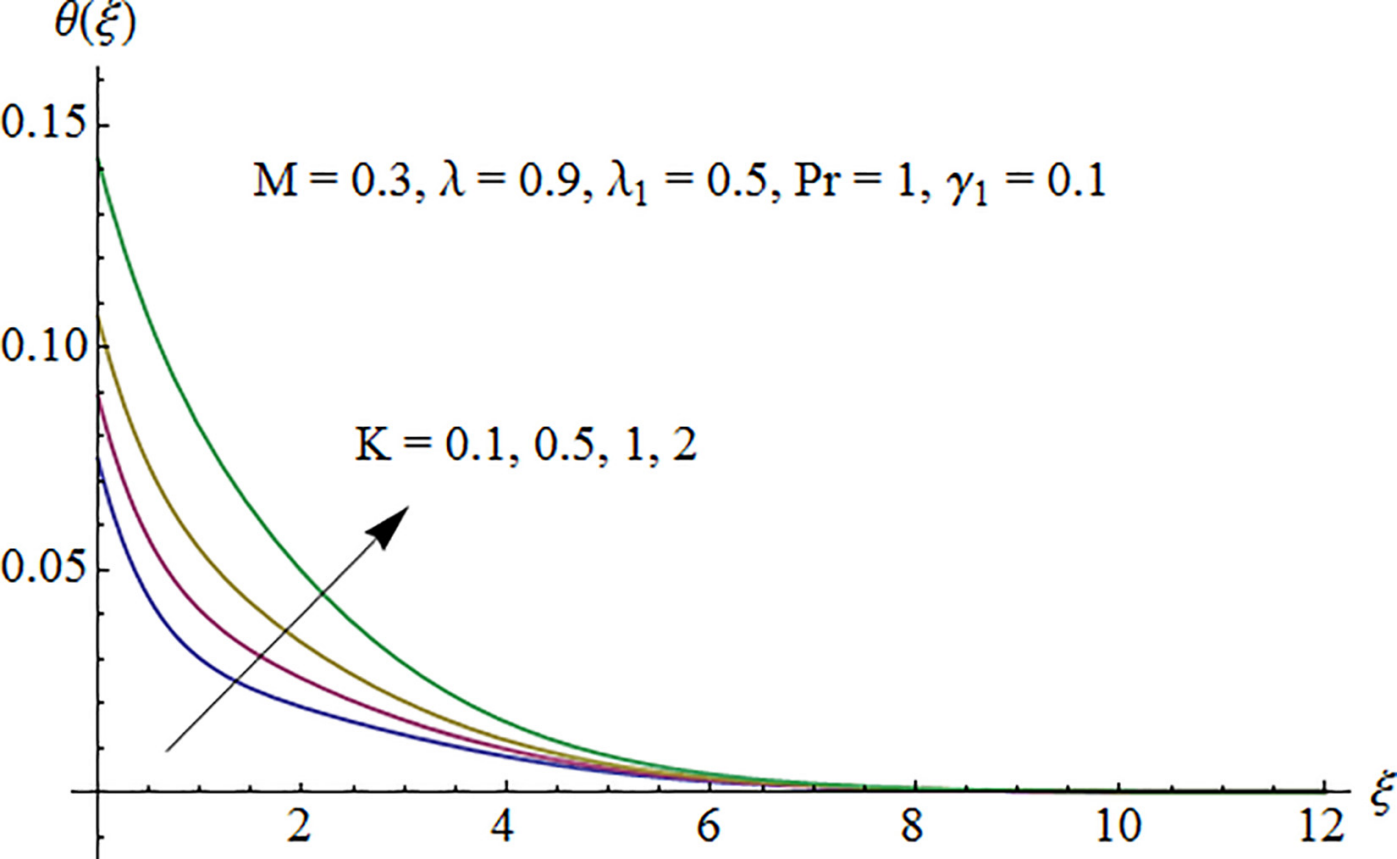
Impact of *K* on temperature.

Temperature profile *θ*(*ξ*) is plotted in Fig 6 to show the effect of Prandtl number Pr. Since the thermal diffusivity decreases by increasing Pr so the temperature decreases. Fig 7 indicates that temperature increases for larger thermal Biot number *γ*_1_ as convective heat transfer coefficient enhances through increasing thermal Biot number *γ*_1_. Fig 8 exhibits variation of curvature parameter *K* on the dimensionless temperature profile *θ*(*ξ*). Enhancement in temperature distribution is observed for larger values of *K*.

### 5.3. Dimensionless concentration profile

**Fig 9:**
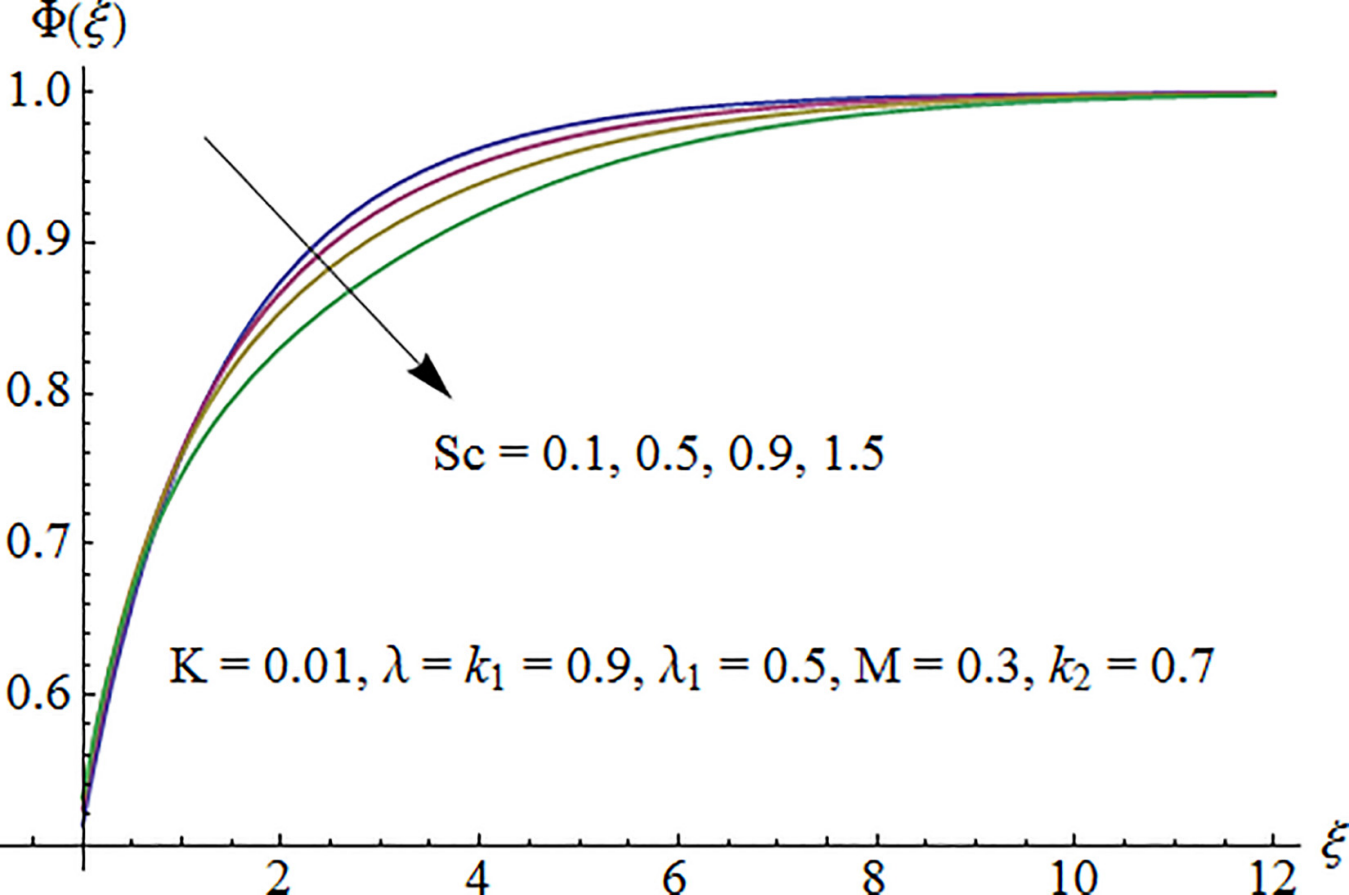
Impact of *Sc* on concentration.

**Fig 10:**
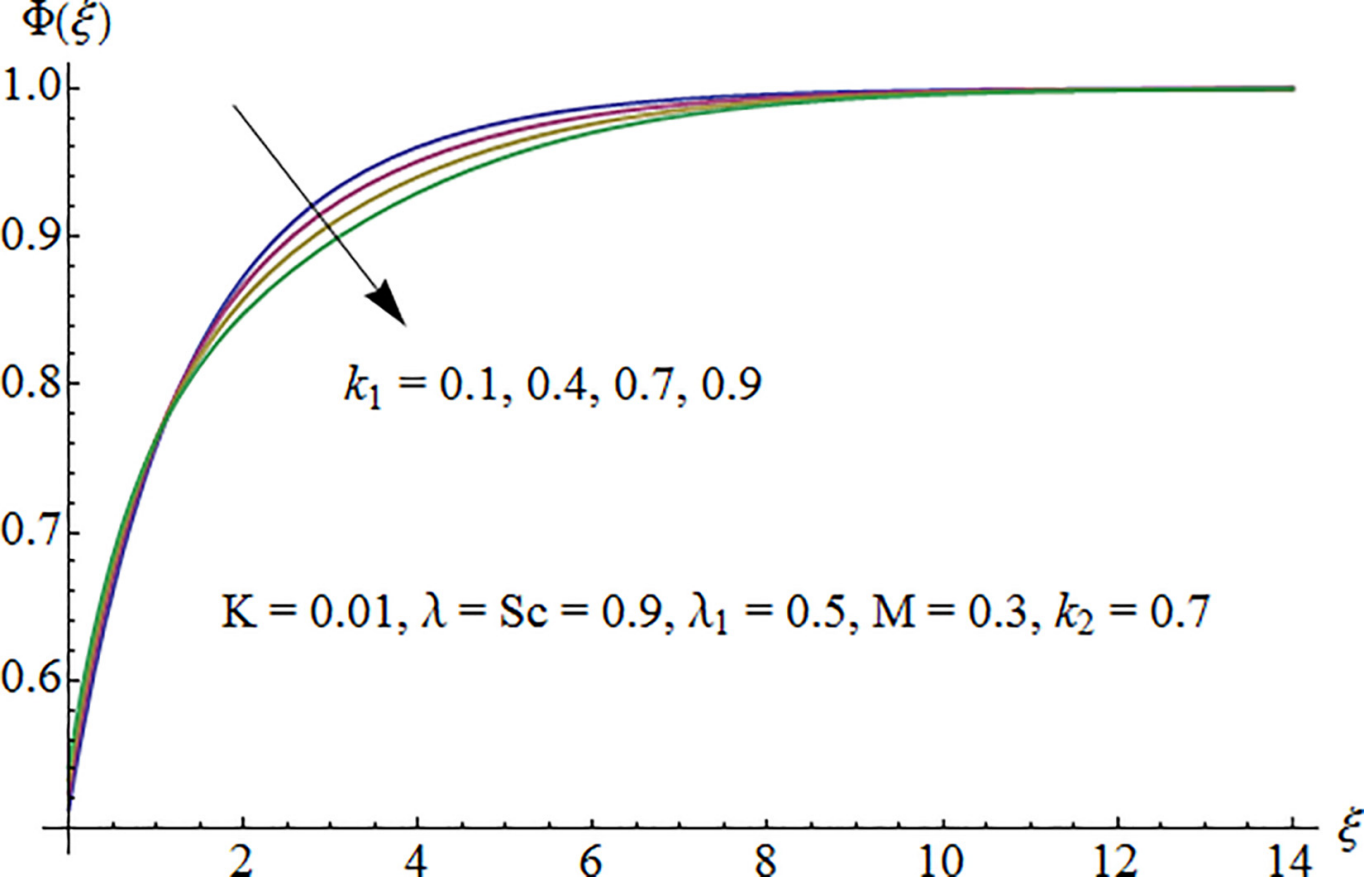
Impact of *k*_1_ on concentration.

**Fig 11:**
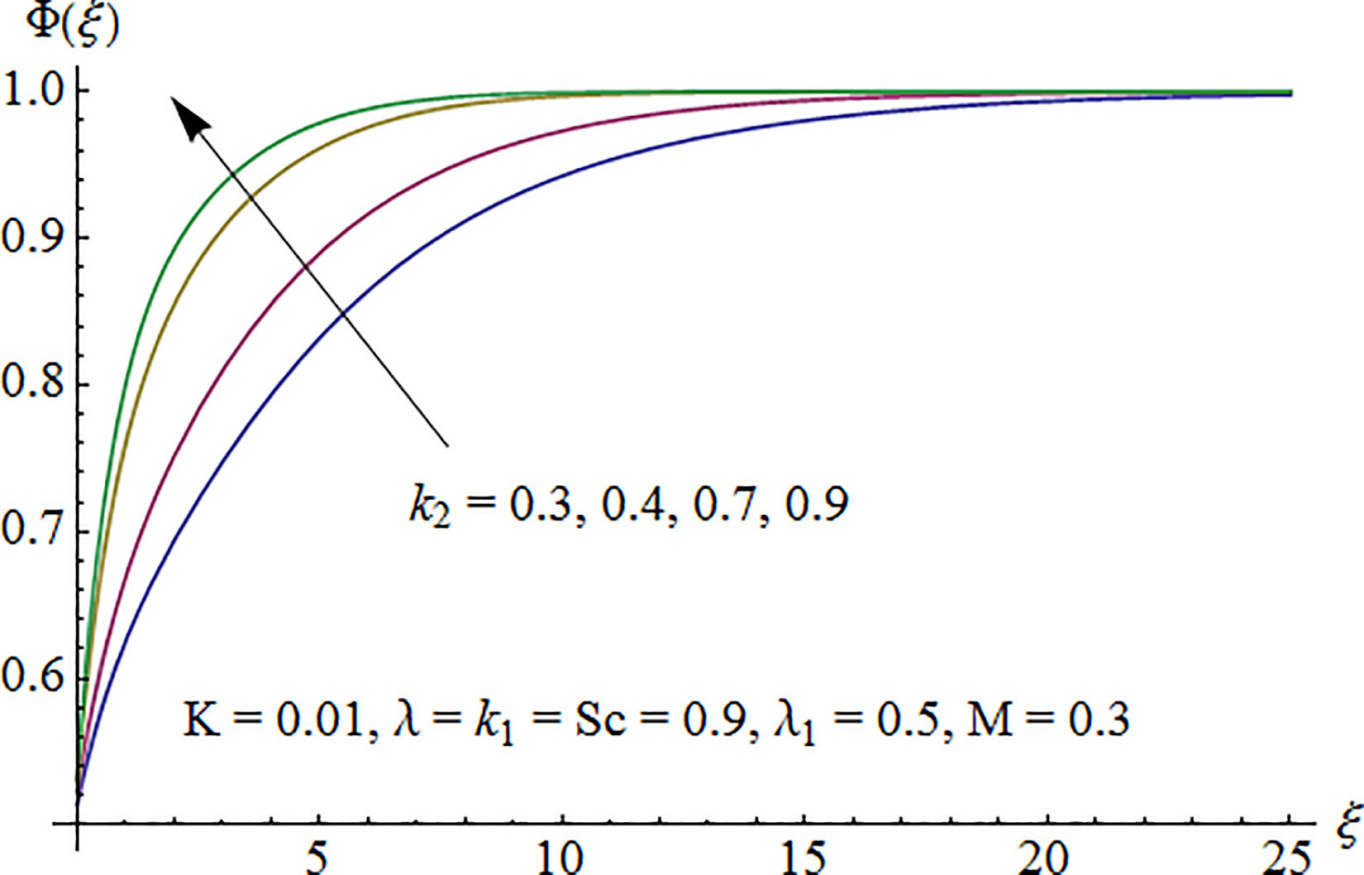
Impact of *k*_2_ on concentration.

Fig 9 shows that concentration profile Φ(*ξ*) is decreasing function of Schmidt number *Sc*. As increase in Sc reduces the mass diffusivity which consequently decrease fluid concentration. Fig 10 depicts the effect of strength of homogeneous reaction parameter *k*_1_ on concentration profile. Fluid concentration decreases due to the consumption of reactants when *k*_1_ is enhanced. Variation of strength of heterogeneous reaction parameter *k*_2_ on Φ is portrayed in Fig 11. Here the concentration profile increases for larger *k*_2_.

### 5.4 Dimensionless pressure profile

**Fig 12:**
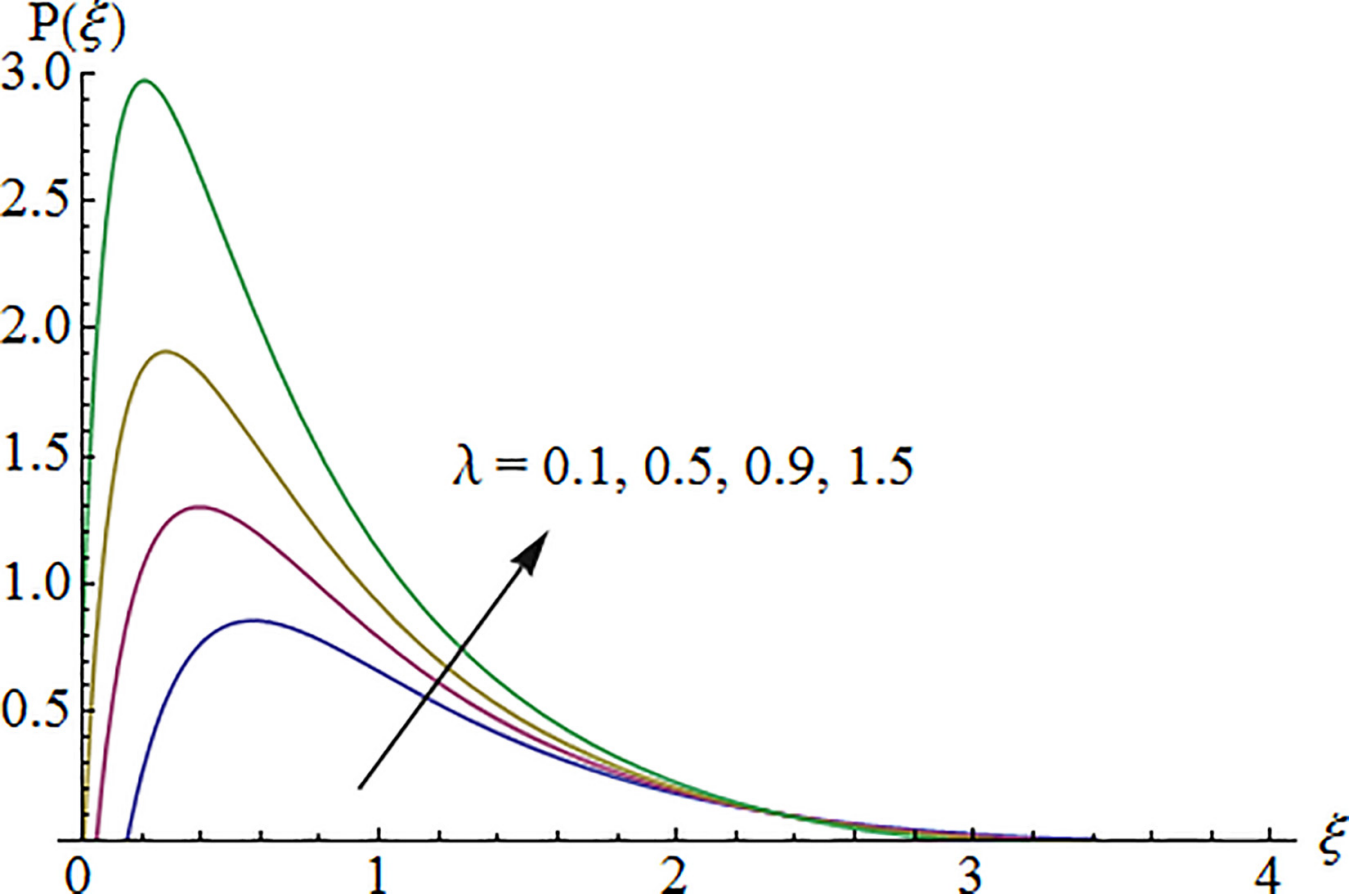
Impact of *λ* on pressure.

**Fig 13:**
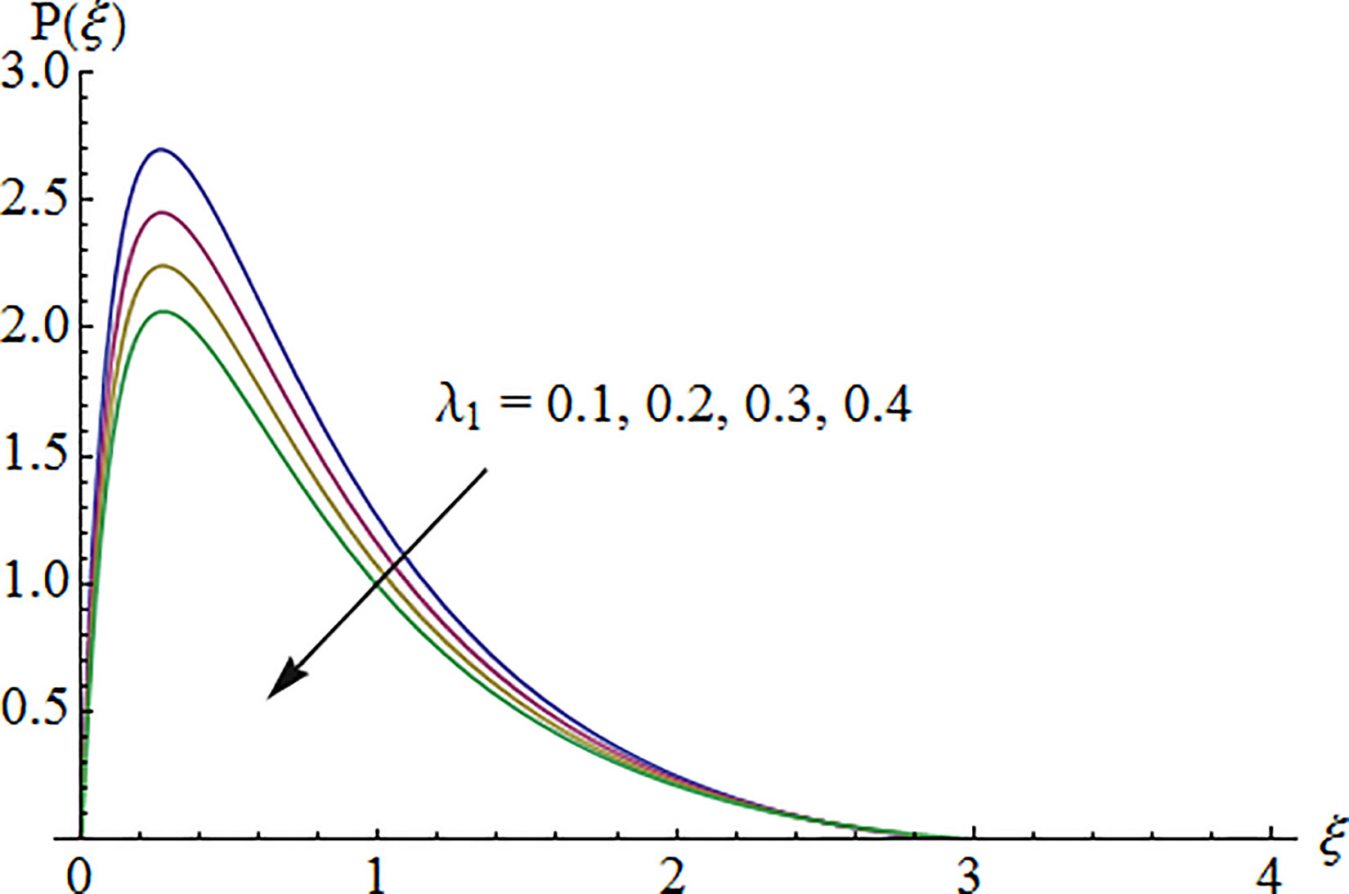
Impact of *λ*_1_ on pressure.

**Fig 14:**
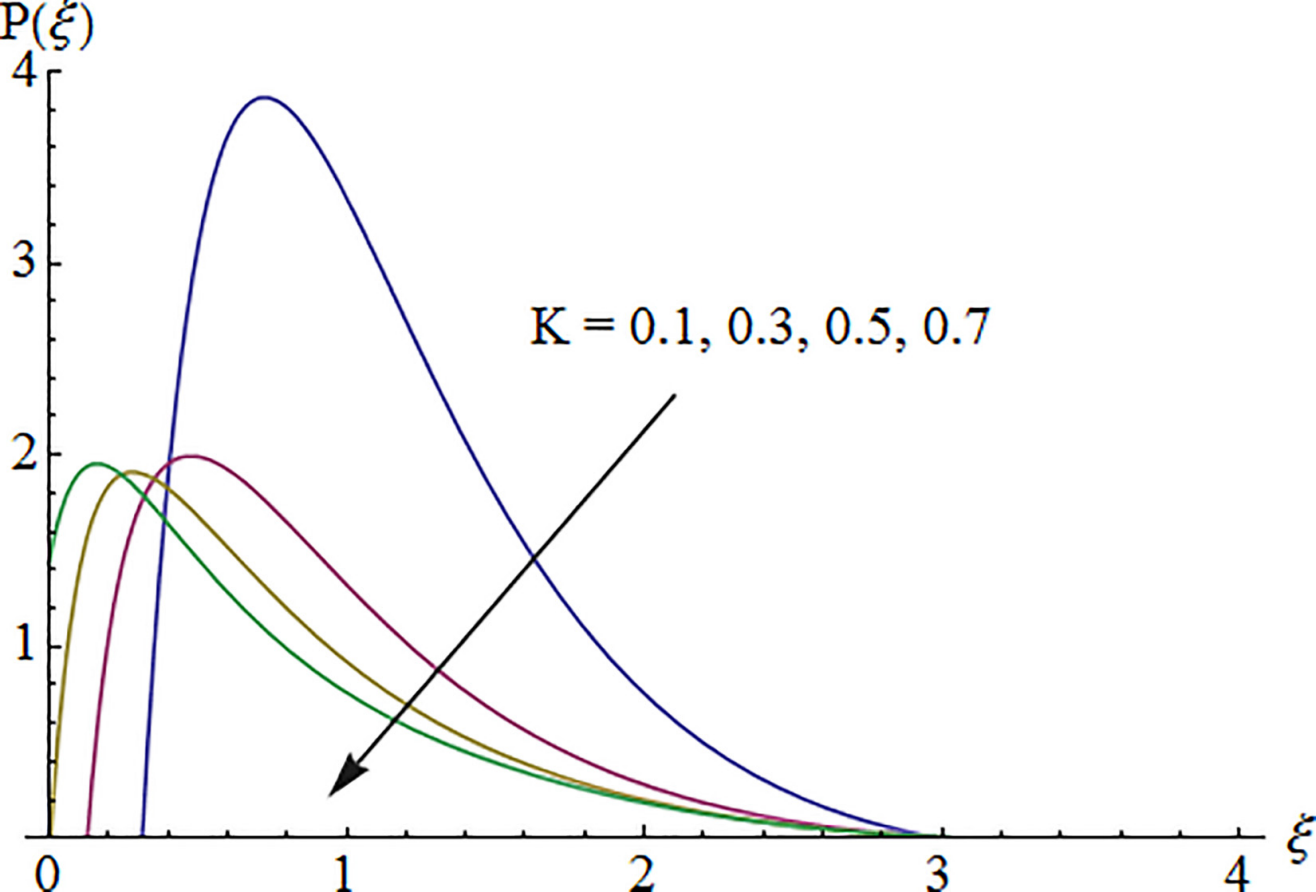
Impact of *K* on pressure.

Figs 12–14 elucidate the variation in pressure profile for increasing values of Deborah number *λ*, ratio of relaxation to retardation times *λ*_1_ and curvature parameter *K*. Here an enhancement in pressure distribution is noted for increasing *λ*. Also pressure is decreasing function of *λ*_1_ and *K*.

### 5.5 Skin friction coefficient

**Fig 15:**
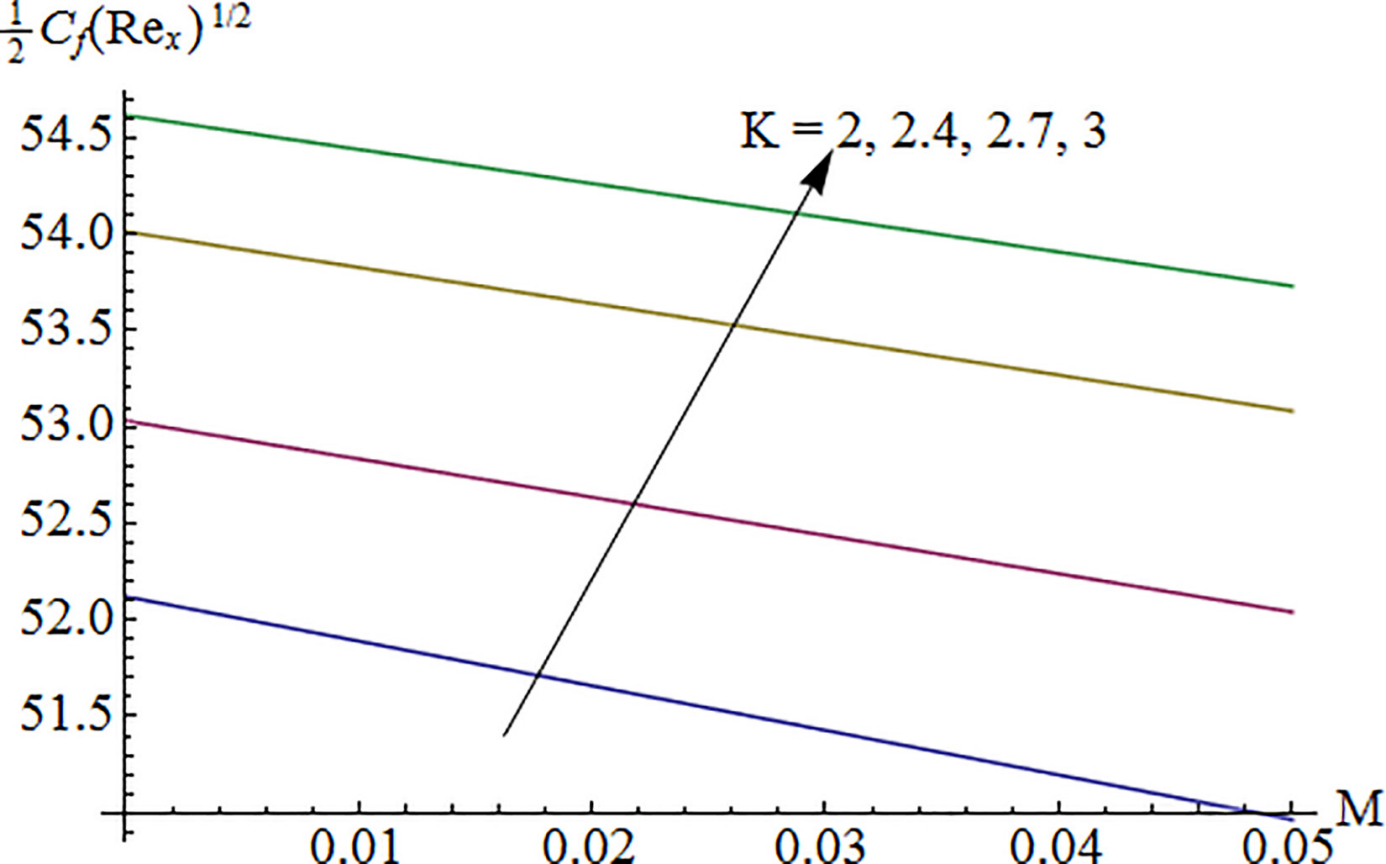
Impact of *K* via *M* on $\frac{1}{2}C_{f}{\left(Re_{x}\right)}^{1/2}.$

**Fig 16:**
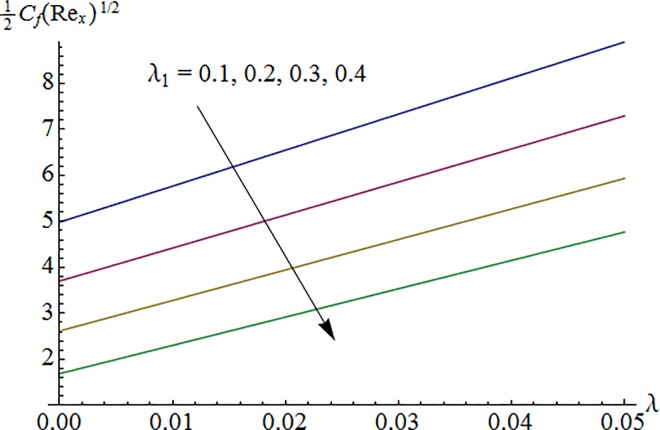
Impact of *λ*_1_ via *λ* on $\frac{1}{2}C_{f}{\left(Re_{x}\right)}^{1/2}.$

Fig 15 shows variation of curvature parameter *K* on surface drag force $\frac{1}{2}C_{f}{\left(Re_{x}\right)}^{1/2}$ against Hartman number *M*. It is noted that skin friction coefficient enhances for larger *K* and it reduces when *M* is increased. Impact of ratio of relaxation to retardation times *λ*_1_ via Deborah number *λ* on surface drag force is illustrated in Fig 16. Here surface drag force decreases for increasing *λ*_1_ while it increases for larger *λ*. Computed results of skin friction coefficient are compared with previously published articles in limiting cases and found in very good agreement (see Table 2).

**Table 2:** Comparison of skin friction coefficient $\frac{1}{2}C_{f}{\left(Re_{x}\right)}^{1/2}$ with previous published articles when *λ*_1_ = 0 = *λ* and *K* = ∞.

M	Hayat et al. [43]	Mabood and Das [44]	Present
1	1.4142	1.4142135	1.4142
5	2.4494	2.4494897	2.4494
10	3.31662	3.31662	3.3166
50	7.14142	7.1414284	7.1414

### 5.6 Nusselt number

**Fig 17:**
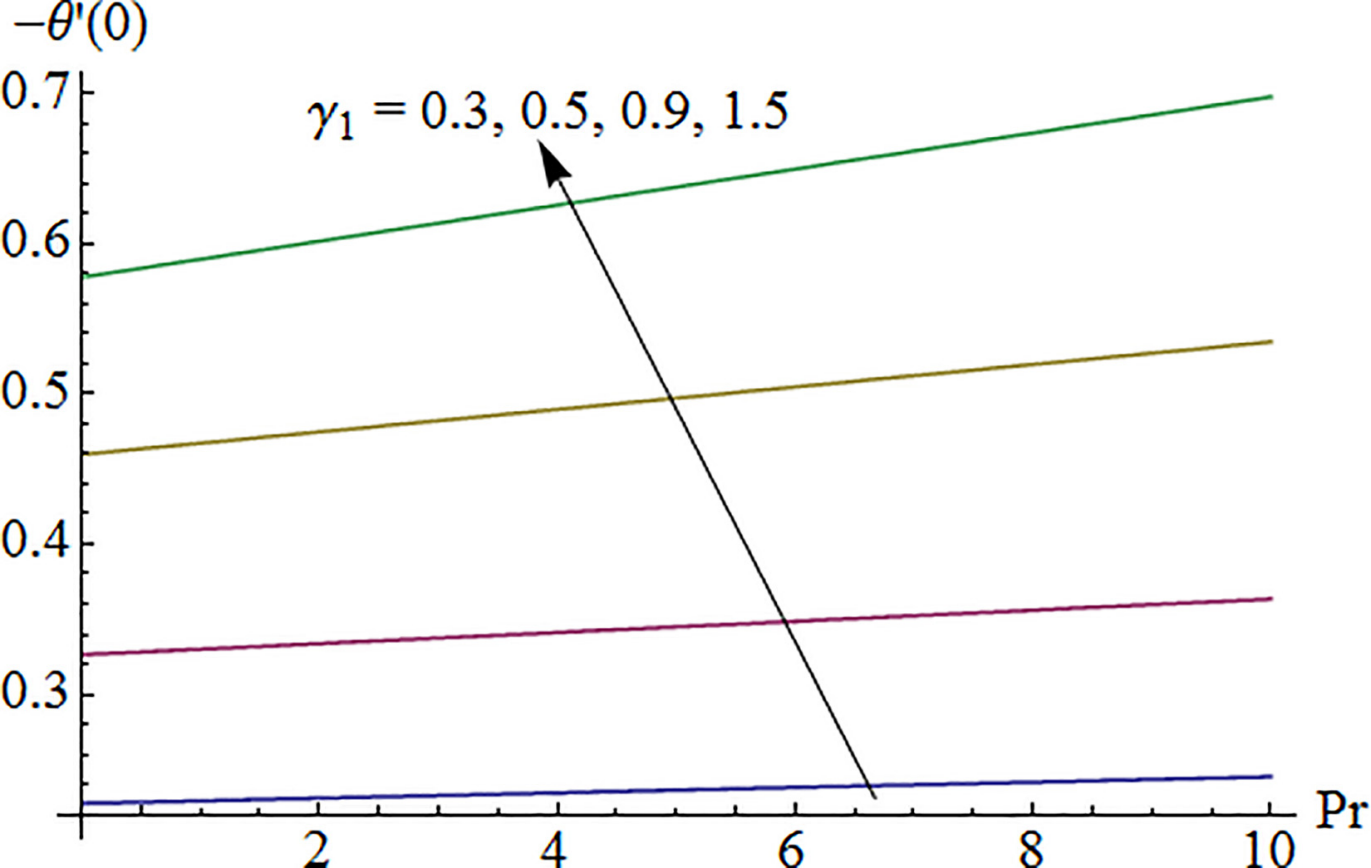
Impact of *γ*_1_ via Pr on –*θ*′(0).

**Fig 18:**
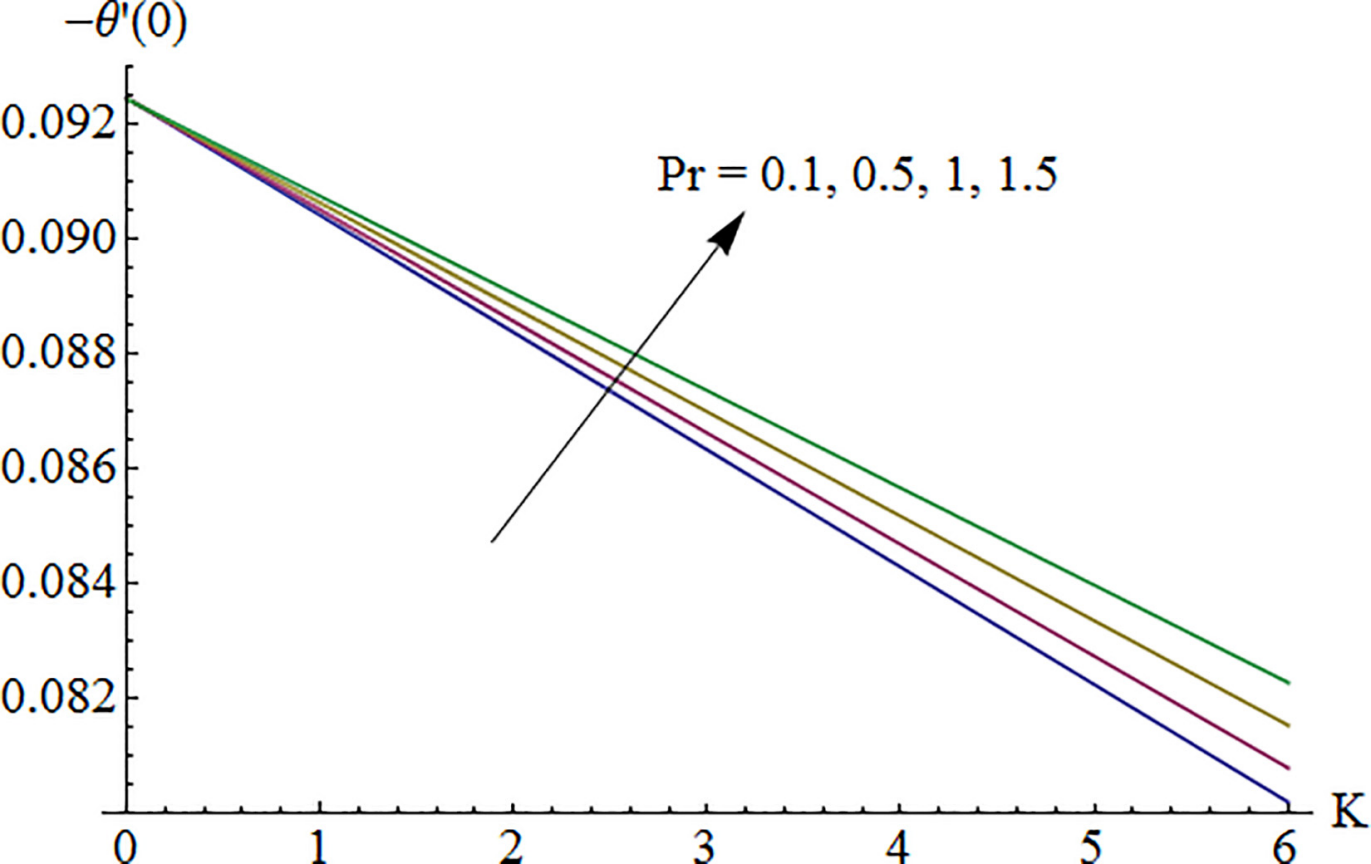
Impact of Pr via *K* on –*θ*′(0).

Impact of Biot number *λ*_1_ on surface heat transfer rate *Nu*(Re_*x*_)^−1/2^ via Prandtl number Pr is shown in Fig 17. It is noted that heat transfer rate enhances for larger values of *λ*_1_ and Pr. Fig 18 illustrates variation of Prandtl number Pr on Nusselt number against curvature parameter *K*. Here surface heat transfer rate decreases as curvature parameter *K* is enhanced while opposite effect is observed for increasing Pr.

### 5.7 Surface concentration

**Fig 19:**
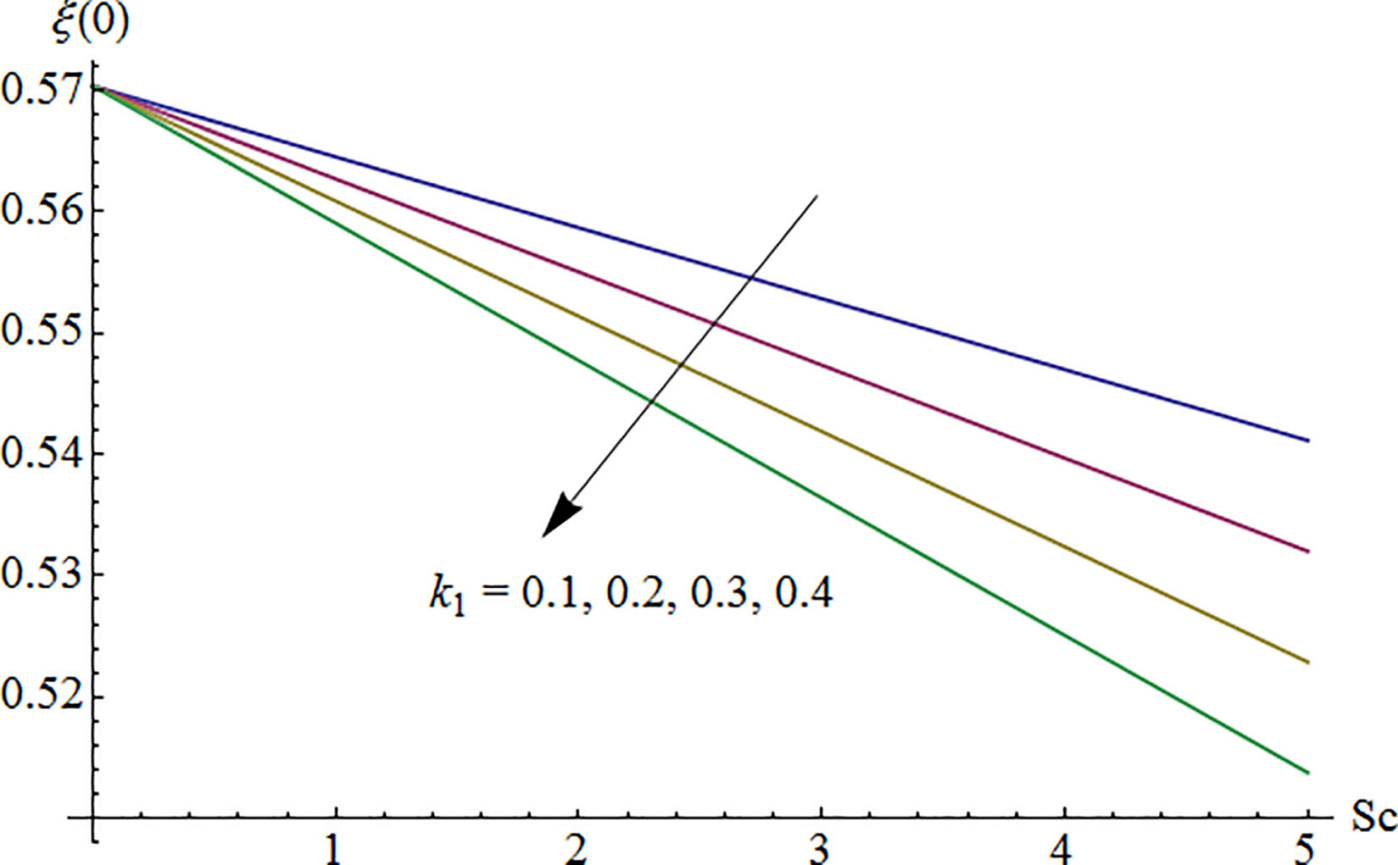
Impact of *k*_1_ via *Sc* on Φ(0).

**Fig 20:**
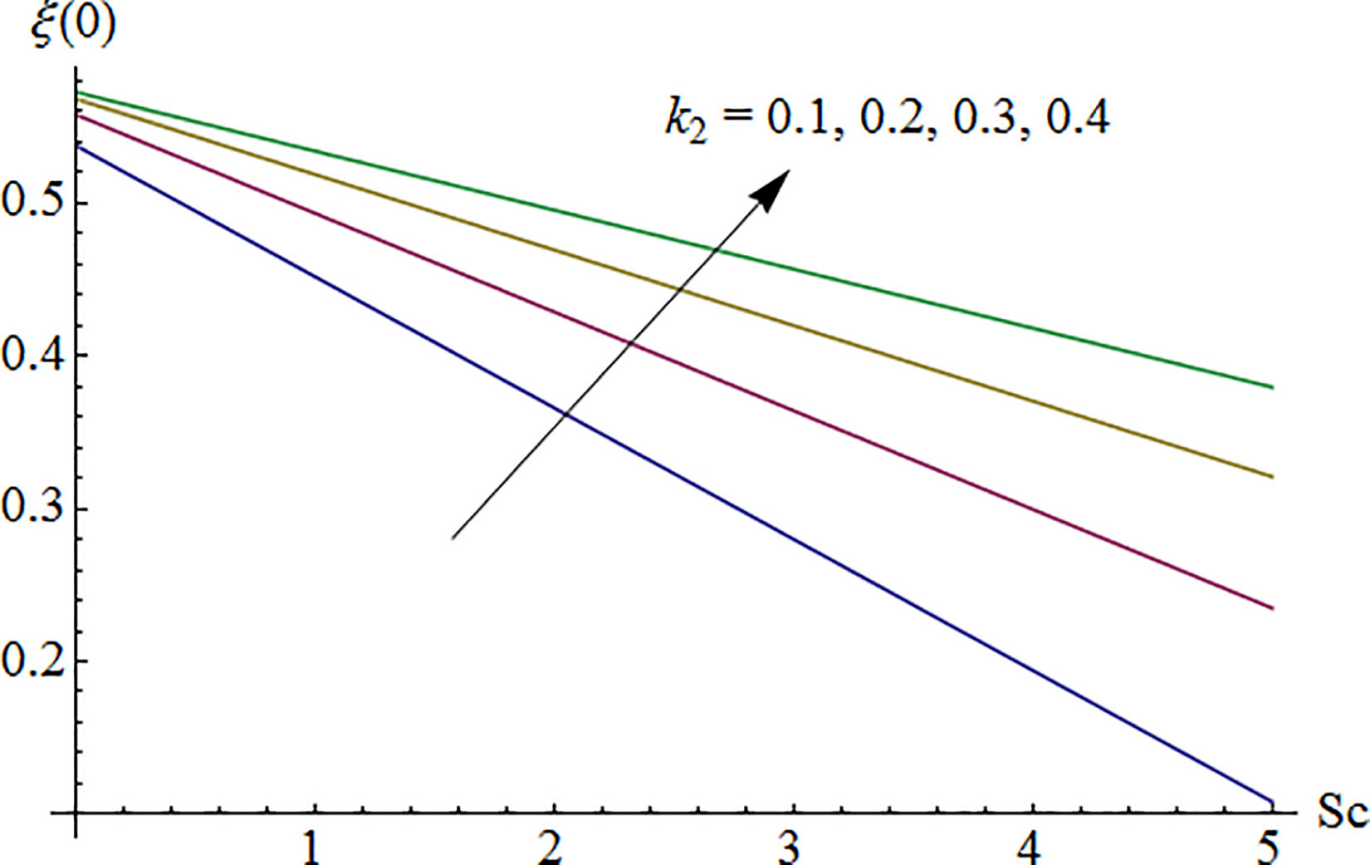
Impact of *k*_2_ via *Sc* on Φ(0).

Variation of homogeneous reaction parameter *k*_1_ on surface concentration Φ(0) against Schmidt number *Sc* is shown in Fig 19. One can see that surface concentration decreases with the increase of *k*_1_ and *Sc*. It is in view of the fact that surface concentration reduces due to the consumption of reactants during chemical reaction. Influence of surface concentration via Schmidt number *Sc* for higher heterogeneous reaction parameter *k*_2_ is depicted in Fig 20. Here surface concentration increases when *k*_2_ is enhanced.

## 6. Concluding Remarks

Effects of homogeneous—heterogeneous reactions in convective flow of Jeffrey fluid due to a curved stretching sheet are studied. The following outcomes are noticed:

- Increase in the values of Deborah number and curvature parameter has similar effects on the velocity in a qualitative sense
- Fluid velocity and temperature enhance for larger curvature parameter.
- The strength of heterogeneous reaction enhances the fluid concentration.
- Pressure distribution has direct relationship with Deborah number.
- Opposite behavior of curvature parameter and Hartman number is seen on the surface drag force.
- Increasing values of Biot number correspond to an enhancement in temperature and Nusselt number.
